# Flexible Nanocomposite Conductors for Electromagnetic Interference Shielding

**DOI:** 10.1007/s40820-023-01122-5

**Published:** 2023-07-07

**Authors:** Ze Nan, Wei Wei, Zhenhua Lin, Jingjing Chang, Yue Hao

**Affiliations:** 1 State Key Discipline Laboratory of Wide Band Gap Semiconductor Technology, School of Microelectronics, Xidian University, 2 South Taibai Road, Xi’an, 710071 People’s Republic of China; 2 Advanced Interdisciplinary Research Center for Flexible Electronics, Xidian University, 2 South Taibai Road, Xi’an, 710071 People’s Republic of China

**Keywords:** Electromagnetic interference shielding, Intrinsically stretchable nanocomposites, Compressible monolith, Low-dimensional materials, Flexible devices

## Abstract

**Highlights:**

- Convincing candidates of flexible (stretchable/compressible) electromagnetic interference shielding nanocomposites are discussed in detail from the views of fabrication, mechanical elasticity and shielding performance.
- Detailed summary of the relationship between deformation of materials and electromagnetic shielding performance.
- The future directions and challenges in developing flexible (particularly elastic) shielding nanocomposites are highlighted.

**Abstract:**

With the extensive use of electronic communication technology in integrated circuit systems and wearable devices, electromagnetic interference (EMI) has increased dramatically. The shortcomings of conventional rigid EMI shielding materials include high brittleness, poor comfort, and unsuitability for conforming and deformable applications. Hitherto, flexible (particularly elastic) nanocomposites have attracted enormous interest due to their excellent deformability. However, the current flexible shielding nanocomposites present low mechanical stability and resilience, relatively poor EMI shielding performance, and limited multifunctionality. Herein, the advances in low-dimensional EMI shielding nanomaterials-based elastomers are outlined and a selection of the most remarkable examples is discussed. And the corresponding modification strategies and deformability performance are summarized. Finally, expectations for this quickly increasing sector are discussed, as well as future challenges.
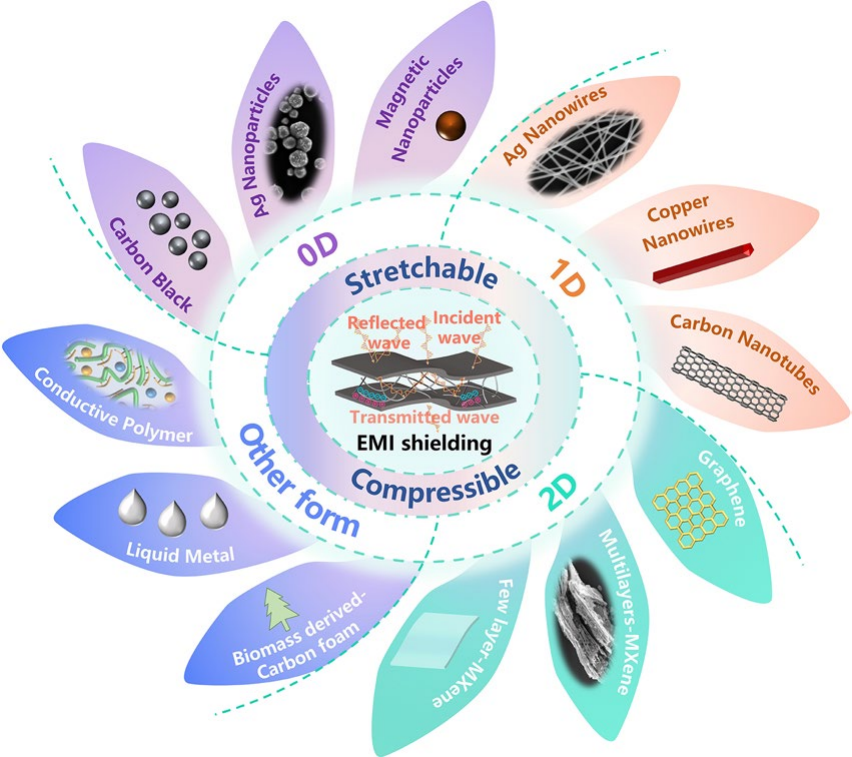

## Introduction

In the information society, electromagnetic (EM) waves, as an important medium for information dissemination, have covered all aspects of production and life in all aspects. With the rapid development of 5G and even 6G wireless communication networks running in the GHz band and the prosperity of portable devices, the problem of EM pollution has risen to an unprecedented level [1].

**Fig. 1:**
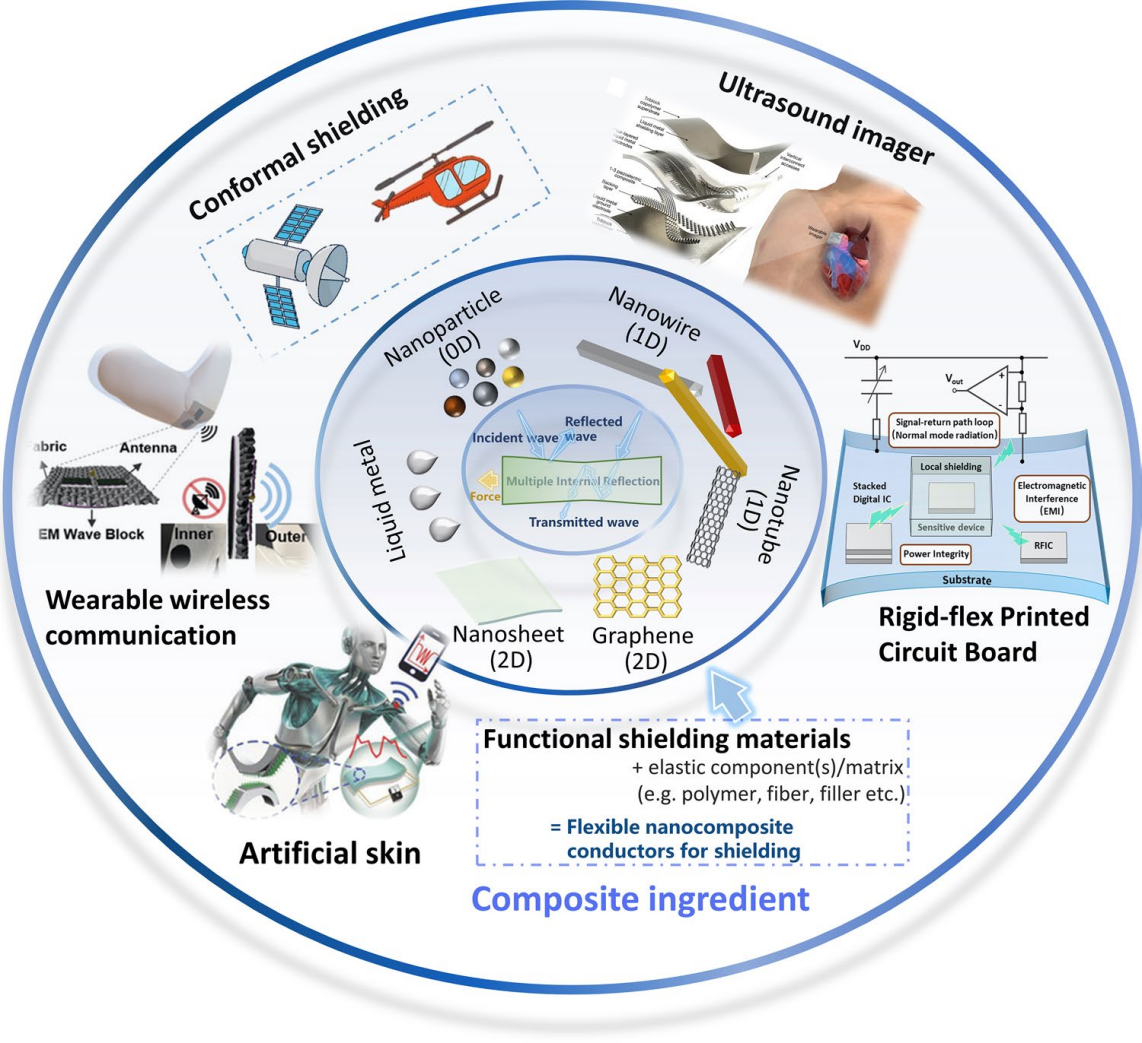
Schematic of the materials and applications of flexible nanocomposite conductors for EMI shielding. Image for “Ultrasound imager”: Reproduced under the terms of the CC-BY Creative Commons Attribution 4.0 International license (https://creativecommons.org/licenses/by/4.0) [280]. Copyright 2023, The Authors, published by Springer Nature. Image for “Artificial skin”: Reproduced with permission [44] [307]. Copyright 2019, Wiley-VCH. Reproduced with permission. Copyright 2018, Wiley-VCH. Image for “wearable wireless communication”: Reproduced with permission [184]. Copyright 2019, Wiley-VCH

Integrated circuits as the cornerstone of the modern information society, with the rapid development of Moore’s law, a single die integrated hundreds of millions of transistors. Meanwhile, on the other hand, three-dimensional heterogeneous microsystem stack integrated a large number of memories, converters, sensors, micro-core processors and other electronic devices. Such high-density integration maximizes the performance of the system, but at the same time introduces serious electromagnetic interference (EMI) between the devices, especially in the system-on-chips (SoC), System-In-Package (SIP), radio frequency integrated circuits (RFIC), and analog circuits (Fig. 1). EMI may cause part of the normal operation of the system failure, so that a significant difficulty challenges the IC dependability [2–5]. In addition, EMI has also produced certain hazards to human health. Studies have shown that a large amount of EM radiation can cause a series of diseases including various cancers, Alzheimer's disease, and reproductive system damage, in addition to making people palpitations and dreamy, increased anxiety, resulting in psychological problems [6].

More demanding specifications have been proposed for flexible wearable devices in recent years due to the advancement of flexible electronics research. Wearables should be able to maintain flexibility, while preserving device performance which is slightly affected by specific deformation circumstances (such as bending, folding, pressing, and stretching) for moving joints and other parts [7–10]. Therefore, EMI shielding films used for wearables are expected to make sure that the EMI shielding efficiency (EMI SE) can still be higher than the minimum value necessary under a particular amplitude of stretching or compression because it is one of the crucial guarantees for the regular operation of sensitive components [11]. The flexible EMI shielding film represented by metallic mesh and ultrathin silver layer sandwiched by oxides film has the characteristics of thinness, transparency and high SE, thus providing optoelectronic applications with robust safety and reliability under RF radiation [12, 13]. Similarly, flexible shielding films prepared with graphene and silver nanowires (AgNWs) as conductive fillers also have the characteristics of flexibility and transparency. At the same time, after replacing the elastic matrix, the elastic shielding film can be formed by matching with the conductive network. Additionally, compared to the previously extensively investigated shielding materials that can be folded and coiled, elastic EMI shielding materials are significantly improved in terms of comfort in joints and other areas of the body where the body moves more frequently, particularly for ultrasound imager and tactile simulation (Fig. 1) [14]. Therefore, the development of flexible wearable devices depends on the creation of stretchable/compressible EMI shielding elastomers. In addition, EMI shielding materials with deformation capabilities suit for conformal surface in modern engineering applications, such as for aircraft and radar [15, 16]. Therefore, a key goal for EM protection is the development of new stretchable and compressible EMI shielding materials with great deformation capacity to protect sensitive objects, enabling improved safety protection for people as well as the regular operation of electronic gadgets [17]. For elastic EMI shielding materials, traditional metal plates, metal nets (etched from metal plates), and other traditional rigid protective materials obviously cannot act as elastic EMI shielding protection.

In recent years, there is a resurgence of interest in low-dimensional nanomaterials such as 0D metal nanoparticles and magnetic nanoparticles, 1D metal nanowires, carbon nanotubes (CNTs), carbon fibers, and other fibrous nanometallic chains, 2D graphene (and its derivants), MXene, and metal nanosheets [18, 19]. These nanomaterials need to form a lattice-like percolation network in order to facilitate the smooth passage of electrons through the junctions as well as the materials themselves. Thanks to the randomly structure of the conductive network, the materials have a certain ability to stretch and compress deformation [20]. Therefore, EMI shielding films, fabrics, and porous materials prepared based on these nanomaterials have many similarities in physical and chemical properties, such as high electrical conductivity and good mechanical properties. Furthermore, the creation of elastic EMI shielding materials also appeals to conductive polymers, liquid metals, and biomass [17, 21]. Elastomers are typically added as matrix to couple with the materials to generate such composite materials as conductive fillers-matrix in order to further improve the mechanical stability and electrical stability of EMI shielding films when stretched and compressed. Below is a thorough review of conductive materials for EMI shielding elastomer.

This review aims to summarize the current research progress on EMI shielding elastomers. According to applied force, we divide the elastic EMI shielding materials into stretchable shielding materials and compressive materials. To better comprehend how elastic EMI shielding films are made, the EMI shielding mechanism (Sect. 2) and mechanical properties of elastomer (Sect. 3) are briefly introduced in the first part. The conductive fillers are analyzed from the material dimension in Sect. 4. In the section, we firstly provide an overview of zero-dimensional nanomaterials, including conductive nanoparticles (e.g., metal nanoparticles-silver nanoparticles (AgNPs), gold nanoparticles (AuNPs), carbon nanoparticles-carbon black (CB), graphite) and magnetic nanoparticles (e.g., ferrites, transition metal nanoparticles), and the resultant elastic composites. Immediately after, one-dimensional nanomaterials-based elastomers, such as AgNWs, copper nanowires (CuNWs) and CNTs, and a few corresponding post-treatment techniques are summarized. Next, two-dimensional nanomaterials-based elastomers, such as graphene and MXene, and some modification strategies for mechanical resilience, stretchability and conductivity will be briefly summarized. Apart from that, several other special and newfangled materials (e.g., conductive polymers, liquid metals, and biomass) will also be reviewed. Eventually, the urgent need for solutions to the issues will be highlighted along with some of the current research directions for stretchable/compressive EMI shielding films.

## Fundamental Mechanisms of EMI Shielding

**Fig. 2:**
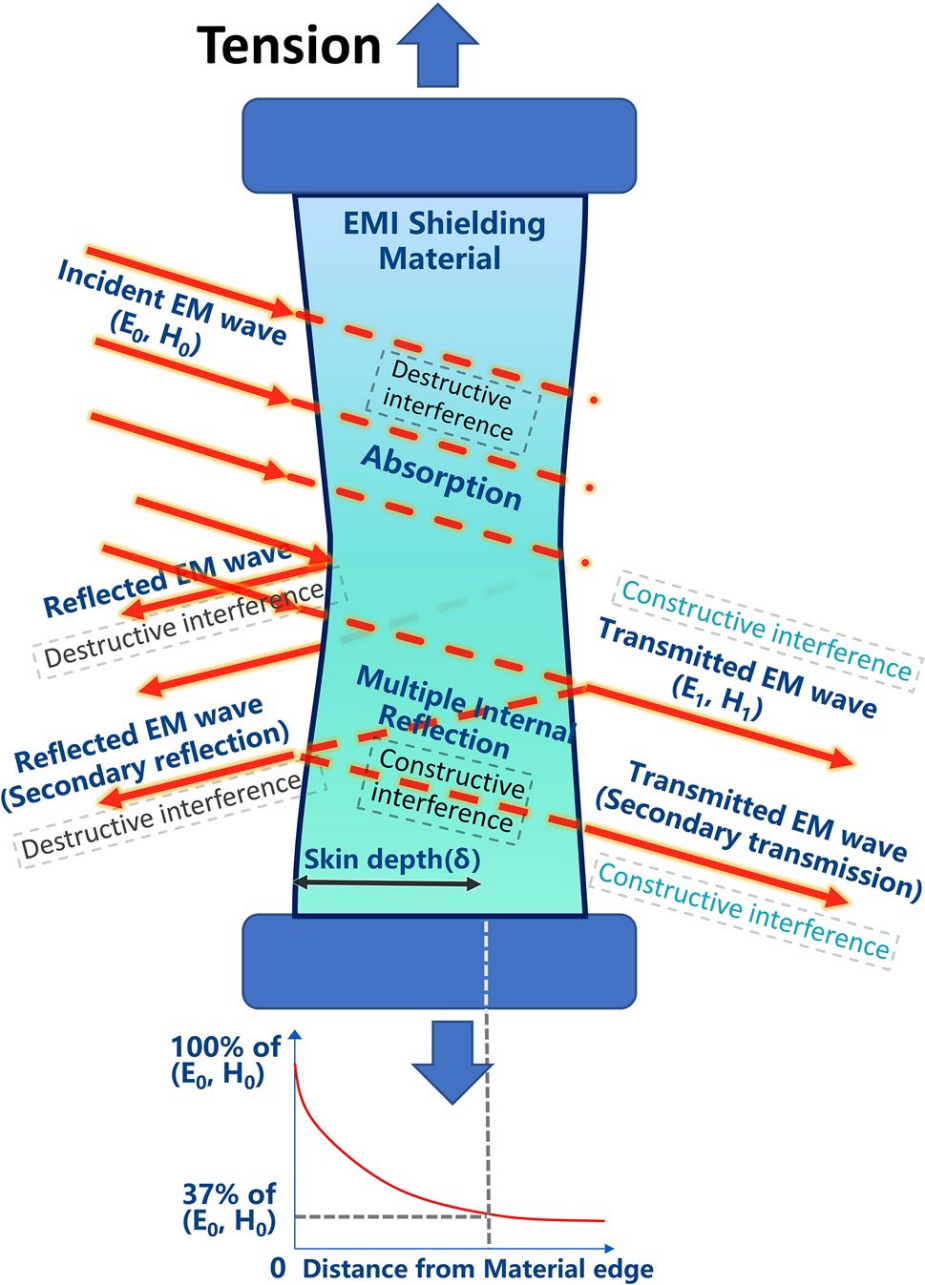
EMW propagation model in EMI shielding material under stretching

When the incident EM waves interact with the surface of the shielding material, four mechanisms of boundary reflection, absorption, internal multiple reflection, and transmission are generated on the surface and inside the material (Fig. 2) [22]. The first three mechanisms will make some attenuation (can be artificially adjusted) of the EM waves, so as to achieve EMI shielding. We generally use the EMI SE to measure the shielding effectiveness of shielding materials against EMI. Defined as the logarithmic ratio of the field measured at the shielded point and the field measured at the same point without shielding, in decibels [12].

1 $${\text{SE }}(dB) = 20\log \left| {\frac{{E_{1} }}{{E_{2} }}} \right| = 20\log \left| {\frac{{H_{1} }}{{H_{2} }}} \right| = 10\log \left| {\frac{{P_{1} }}{{P_{2} }}} \right|$$

where *E*, *H*, and *P* are electric field strength, magnetic field strength, and EM wave power, respectively. At the same time, combined with the mechanism of EM wave action in the material, Schelkunoff’s formula states that the EMI SE_T_ is an important indicator of the attenuation ability of the material, which consists of reflection loss, absorption loss, and multiple reflection loss [23]. See below for details:

2 $${\text{SE}}_{T} (dB) = {\text{SE}}_{R} + {\text{SE}}_{A} + {\text{SE}}_{M}$$

When EM waves pass through the shielding material, the dipole in the material interacts with the EM field to convert the EM wave energy into heat, then dissipating the caused [24]. From the formula, the absorption loss is related to the thickness of the material, magnetic permeability, electrical permeability, and the frequency of EM waves. The absorption of EM waves is caused by dielectric and magnetic losses [25]. Therefore, the larger the loss factor, the more favorable the absorption of EM waves, where *d* denotes the material thickness, *f* denotes the incident wave frequency, *μ* denotes the magnetic permeability, and* σ* denotes the electrical conductivity.

3 $$SE_{A} (dB) = 8.7d\sqrt {\pi f\mu \sigma }$$

However, when the impedance of the shielding material does not match the transmission impedance of EM waves in space, the charged particles of the shielding material will interact with the electric field and be reflected by the interface, resulting in reflection loss [26]. As shown in Eq. (4), the higher conductivity and lower the permeability of the materials, resulting in greater reflection loss. For example, the reflection loss of traditional metal materials (copper plate, silver plate, etc.) is high, and it can be assumed that its shielding mechanism for EM waves is almost totally reflection.

4 $$SE_{R} (dB) = 39.5 + 10\log \sqrt {\frac{\sigma }{2\pi f\mu }}$$

There is also a loss mechanism, when the EM waves in the shielding body at multiple interfaces inside the repeated reflection and transmission caused by the loss of EM waves [20]. It is known that the skin effect that high-frequency EM waves will be concentrated on the surface of the material. If the thickness is much greater than the skin depth* δ*, multiple reflections can be ignored, while if the thickness is close to or even less than *δ*, then multiple reflections must be considered.

5 $$SE_{MR} = 20\log \left[ {1 - \exp \left( {\frac{2d}{\delta }} \right)} \right]$$

In addition, it is currently one of the research hotspots in the field of EMI shielding to improve the multiple reflection loss inside the material and increase the dissipation of EM waves by reflecting them multiple times at the reflection interface provided inside the shielding materials [27]. It is worth noting that in some scenarios, the EM waves are supposed not to be mostly reflected and deteriorate the EM environment in the surrounding space, hence expecting the EMI shielding material to increase the absorbing loss and decrease the reflecting loss [28].

In general, the total shielding efficiency of electromagnetic waves (SE_T_) is subject to the combined effects of reflection loss (SE_R_), absorption loss (SE_A_), and multi-reflection loss (SE_MR_), which arise from the behavior of mobile charge carriers, the presence of electric (or magnetic) dipoles, and the interactions of waves with various surfaces or interfaces, respectively [29, 30]. Notably, strain-induced changes of electrical properties and composite microstructure and monolithic thickness jointly impact the overall EMI SE in deformable shielding armors. When subjected to deformation, the strengthening or weakening of the conductive circuit has a direct impact on the movement of charge carriers, leading to a change in conduction loss. Simultaneously, the change of thickness affects the propagation path of EM wave in the lossy medium [31]. In the process of propagation, the interaction with the electrical (magnetic) dipole will generate dielectric or magnetic losses, resulting in more absorption dissipation. And eventually residual EM waves dissipate in the form of heat energy. Moreover, the 3D microstructures introduce abundant conductive surfaces and facilitate multiple reflection/scattering and subsequent absorption of the EM waves inside the conductive network when stretching [23]. The individual contribution of the aforementioned factors governing the shielding performance in flexible matrices can vary significantly, thereby necessitating a meticulous assessment tailored to the specific material architecture.

Experimentally, the EMI SE is typically determined by measuring the scattering parameters, S11 and S21, using a vector network analyzer (VNA), with their relationship expressed by Eq. (6):

6 $${\text{SE}}(dB) = - 10\log \left( {\frac{1}{{\left| {S_{21} } \right|^{2} }}} \right)$$

When an electromagnetic (EM) wave encounters the surface of a shielding material, the total of its reflection coefficient (*R*), absorption coefficient (*A*), and transmission coefficient (*T*) must be conserved [23]. They can be calculated by scattering parameters and expressed as:

7 $$R + A + T = 1$$

8 $$R = \left| {S_{11} } \right|^{2}$$

9 $$T = \left| {S_{21} } \right|^{2}$$

In the field of wearable devices, in addition to meeting the requirements of EMI shielding, shielding materials also need to pursue the ‘thin, light’ (thin-shielding material thickness is small; light-low density) to improve the comfort of wearable devices. In addition, in aerospace, integrated circuits, and other fields, lightweight materials can effectively reduce the overall weight, saving energy and space [32]. In order to measure the performance of the materials and fully consider the influence of thickness and density on the SE of the materials, we defined the following three specific shielding effectiveness as follows [33]:

10 $$SSE = \frac{EMI\;SE}{\rho }(dB\;cm^{3} g^{ - 1} )$$

11 $$SE/t = \frac{EMI\;SE}{d}(dB/mm)$$

12 $$SSE/t = \frac{EMI\;SE}{{\rho \cdot d}}(dB\;cm^{2} g^{ - 1} )$$

Specific shielding effectiveness (SSE) combines three key parameters (SE, thickness *d*, and density *ρ*) and is important in measuring the EMI SE of lightweight and thin materials. The larger the value, the thinner and lighter the material is, and at the same time the better the shielding effectiveness itself [30]. This parameter has been widely used in the field of porous EMI shielding materials and ultrathin EMI shielding materials.

For thin stretchable shielding films, the conductivity is generally measured by the square resistance (*R*_sq_). A theoretical analysis of the relationship between the EMI SE in a high frequency (higher than 30 MHz) and *R*_sq_ can be summarized with an empirical formula as follows [34–36]:

13 $${\text{SE}}\; = \;20\log \left( {1{ + }\frac{{Z_{0} }}{{2R_{{{\text{sq}}}} }}} \right)$$

where *Z*_0_ is the impedance of free space (377 $\Omega$).

## Mechanical Properties of Elastomer

The mechanical properties of elastomer are related to the response of an object made of that elastomer to an applied force, called loading, thus determining the specific application and deformable limit [37, 38]. The most important properties include strength, hardness, ductility, and fracture toughness. Note that the elastomers have the capacity of returning to their original state and size after stress relaxation has been removed; however, materials that are merely elastic are not elastomers [39]. However, elastomers may fracture (for stretchable elastomers) or collapse (for compressible elastomers) when subjected to loads that are beyond their tolerance. This is a crucial problem in the field of elastic electronics, as standards are not yet fully established and the statistical repeatability of newly reported materials/devices is frequently uncertain.

**Fig. 3:**
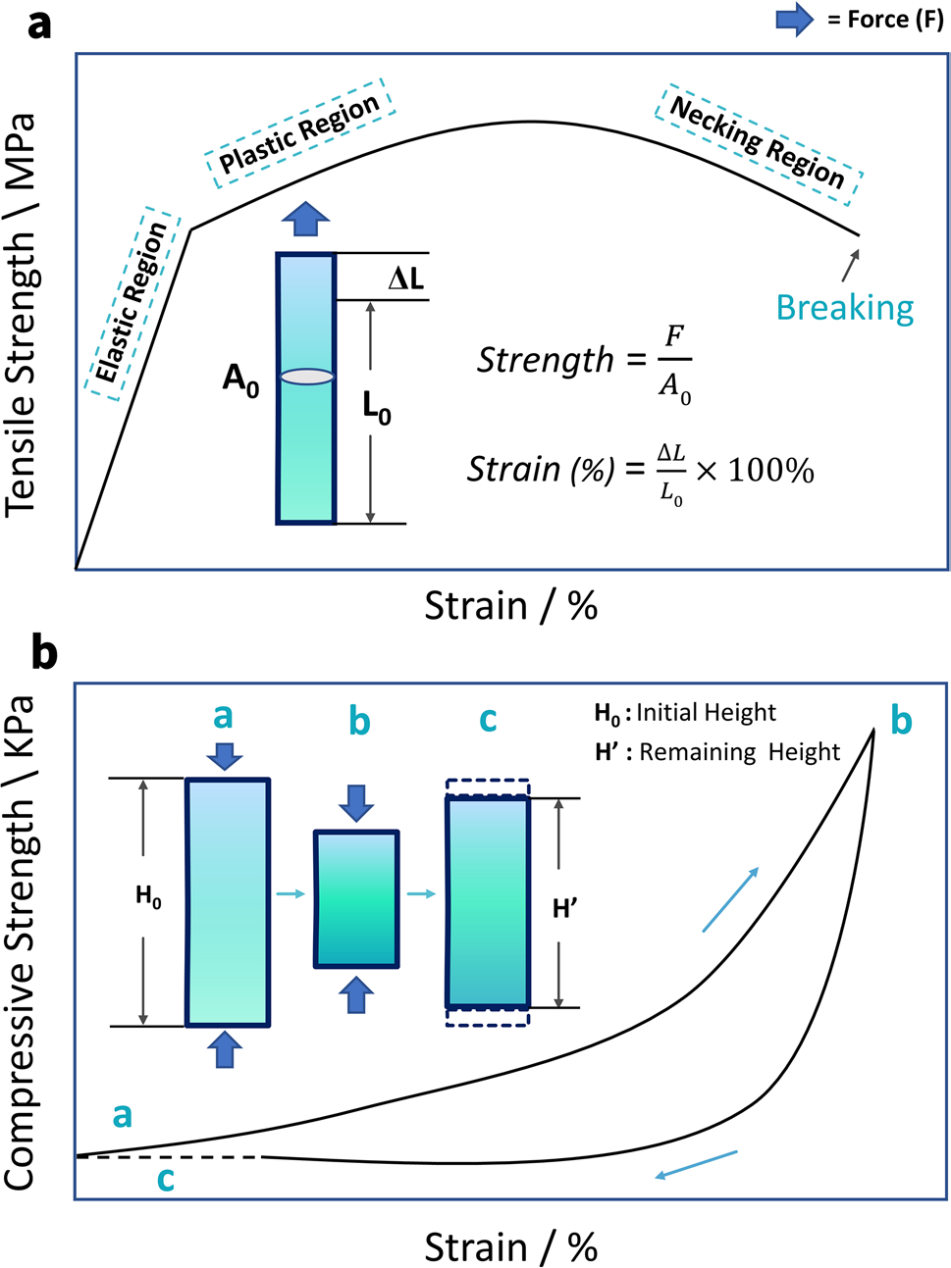
**a** Tensile strength–strain of stretchable materials. Inserts are representation of tensile load to a wire and corresponding equations. **b** Compressive strength–strain curves of compressible materials. Inserts are the schematic diagram showing the compression-recovery process

Elastomers can be broadly divided into stretchable and compressible elastomers in terms of force direction and application scenarios. Tension is a type of loading in which two sides of an object are pulled apart. When tension is applied, a material that resembles a regular wire, for instance, deforms. Thus, in this scenario, the stress is a vector simply given by strength = *F/A*, with a unit measure of N m^−2^, termed the pascal (Pa). Deformation under stress is measured by a quantity called strain (*ε*), which is defined as the object's reaction to the tension. Strain, in percentage terms, for a wire under tension stress is the percentage of elongation over the initial length, or strain (%) = * ΔL*/*L*_0_ × 100% (Fig. 3a) [40]. The straight line segment of this curve represents an elastic deformation, where the shape of the object is restored to its pre-stress condition when the stress is removed (Hooke's law) [41]. Young’s modulus is a measure of a material’s stiffness that is equal to the gradient of the line of elastic region. As the tensile force exceeds the range of elastic deformation, the deformation of the material turns into plastic deformation, i.e., the material cannot recover its initial length after stretching. After this, the material shows signs of necking until it breaks completely [42].

Compression is the inverse action of tensile loading, and it is accomplished by forcing the material together. Compressible elastomers may not achieve a return to 100% of their original height during compression-release testing due to partial collapse and deformation of the internal structure [43]. This is largely dependent on differences in skeleton flexibility due to different processes of compressible material preparation. Also, for lightweight compressible 3D monoliths such as sponge, foam and aerogel, the compression strength is generally low, remaining only in the KPa range. In this case, the ratio of the remaining height (*H*’) to the initial height (*H*_0_) after different number of compression cycles at different stresses is generally used to reflect the fatigue resistance of compressible materials (Fig. 3b) [44].

14 $${\text{Height}}\;{\text{retention}}\;{\text{(\% )}}\;{ = }\;\frac{H^{\prime}}{{H_{0} }} \times 100\%$$

## EMI Shielding Materials

**Fig. 4:**
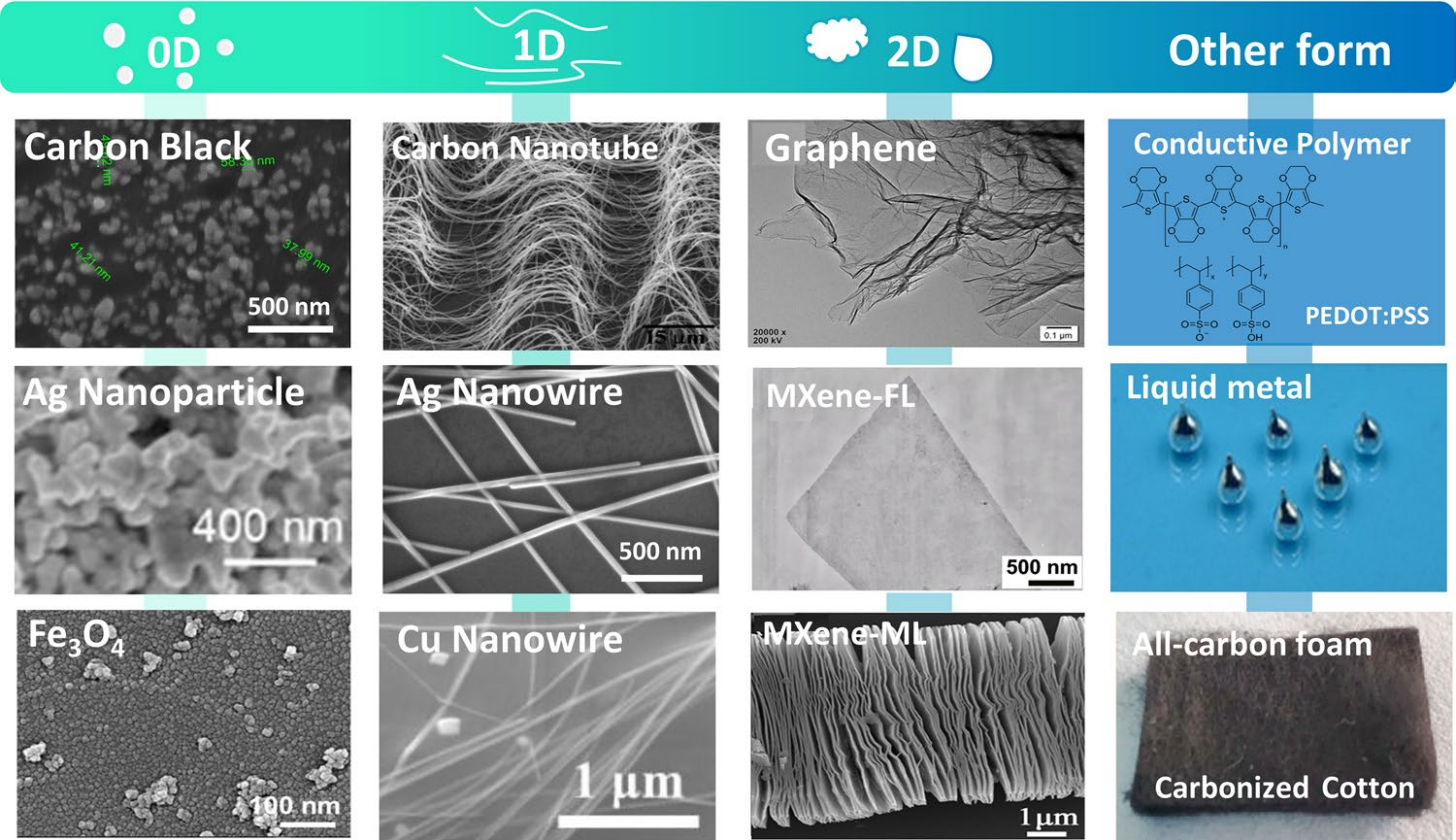
Commonly used electronic fillers for elastic EMI shielding materials. Image for “Carbon Black”: Reproduced with permission [72]. Copyright 2021, Elsevier Ltd. Image for “Ag Nanoparticle”: Reproduced with permission [64]. Copyright 2019, Wiley–VCH. Image for “Fe3O4”: Reproduced with permission [91]. Copyright 2021, American Chemical Society. Image for “Carbon Nanotube”: Reproduced with permission [281]. Copyright 2012, Wiley–VCH. Image for “Cu Nanowire”: Reproduced with permission [282]. Copyright 2022, Elsevier B.V. Image for “graphene”: Reproduced with permission [283]. Copyright 2018, American Chemical Society. Image for “MXene-few-layer structure (FL)”: Reproduced under the terms of the CC-BY Creative Commons Attribution 4.0 International license (https://creativecommons.org/licenses/by/4.0) [106]. Copyright 2021, The Authors, published by Springer Nature. Image for “MXene-multilayer structure (ML)”: Reproduced with permission [284]. Copyright 2019, The Society of Powder Technology Japan. Image for “Liquid metal”: Reproduced with permission [260]. Image for “All-carbon foam”: Reproduced with permission [273]

The EMI SE of deformable shielding composites mainly depend on the components and microstructures of electronic fillers [45]. In general, materials with superior shielding effectiveness, such as good EM wave reflection and dissipation capacity, as well as inherent softness and mechanical deformability, are potentially excellent electronic filler. The foregoing can broadly summarize the harmful EM interference and thoroughly reveal the fundamental mechanisms of EMI shielding. In this section, the research highlights on the design of: (i) zero-dimensional (0D), (ii) one-dimensional (1D), and (iii) two-dimensional (2D) nanomaterials are discussed below (Fig. 4).

### 0D Materials

Compared with conventional metallic materials (e.g., metal sheets, metal blocks, and metal meshes, etc.), 0D materials developed in recent years have become more prospective alternatives for EMI shielding on account of their large specific surface area, less thickness, low cost and excellent compatibility with other conductive fillers. [46]. Depending on the components of fillers, the functional 0D materials used for EMI shielding can be divided into two categories: One is the conductive nanoparticles, and the other is the magnetic nanoparticles.

#### Conductive Nanoparticles

##### Metal Nanoparticles

Metal nanoparticles with diameter 20–200 nm offer great potential as a low-cost and efficient alternative to expensive and high-density conventional metallic materials used for EMI shielding owing to their ultrahigh electrical conductivity, super-large specific surface area, as well as low cost [47]. Owing to their high electronic conductivity, metal nanoparticles, including Ag, Au, and others, can be used for EMI shielding as dominant conductive materials in polymer composites, which are employed in both industry and scientific research for EMI shielding [48–51]. Furthermore, the broad processing window and reduced number of process steps available for metal nanoparticle production confer a high degree of simplicity and scalability on their production. Meanwhile, due to the low preparation cost and mild reaction conditions of the wet chemical process, it has become the main preparation method for the synthesis of metal particles [52–57].

Considering the filler concentration and deformability of the composites, this multiphase composite system is prepared by compounding the polymer matrix with metal nanoparticles. The commonly used assisted matrixes include polyurethane (PU) [58–60], poly(dimethyl siloxane) (PDMS) [61, 62], Poly(styrene-butadiene-styrene) (SBS) [63], poly(styrene-co-ethylenebutylene-co-styrene) (SEBS) [64], cellulose nanofibers (CNF) [60, 65], melamine–formaldehyde foam, etc. [61, 66]. With regard to the construction of the dense conductive network, metal ions in solution could be firstly reduced to form metal nanoparticles, and then be uniformly wrapped on any surface so that the composite could be rendered abundantly conductive to be employed in EMI shielding [58–60, 63, 64, 66]. The sufficient metal nanoparticles can offer numerous mobile charge carriers, which boost enormously conductivity and result in the massive ohmic and eddy current losses for a very high EMI dissipation [67, 68]. Moreover, when the residual EM wave enters inside the composite film, the large conductivity mismatch between conductive metal nanoparticles and insulating polymer matrixes are beneficial to the polarization relaxation and charge accumulation which help to dissipate the EM wave by interfacial effect [59, 69].

**Fig. 5:**
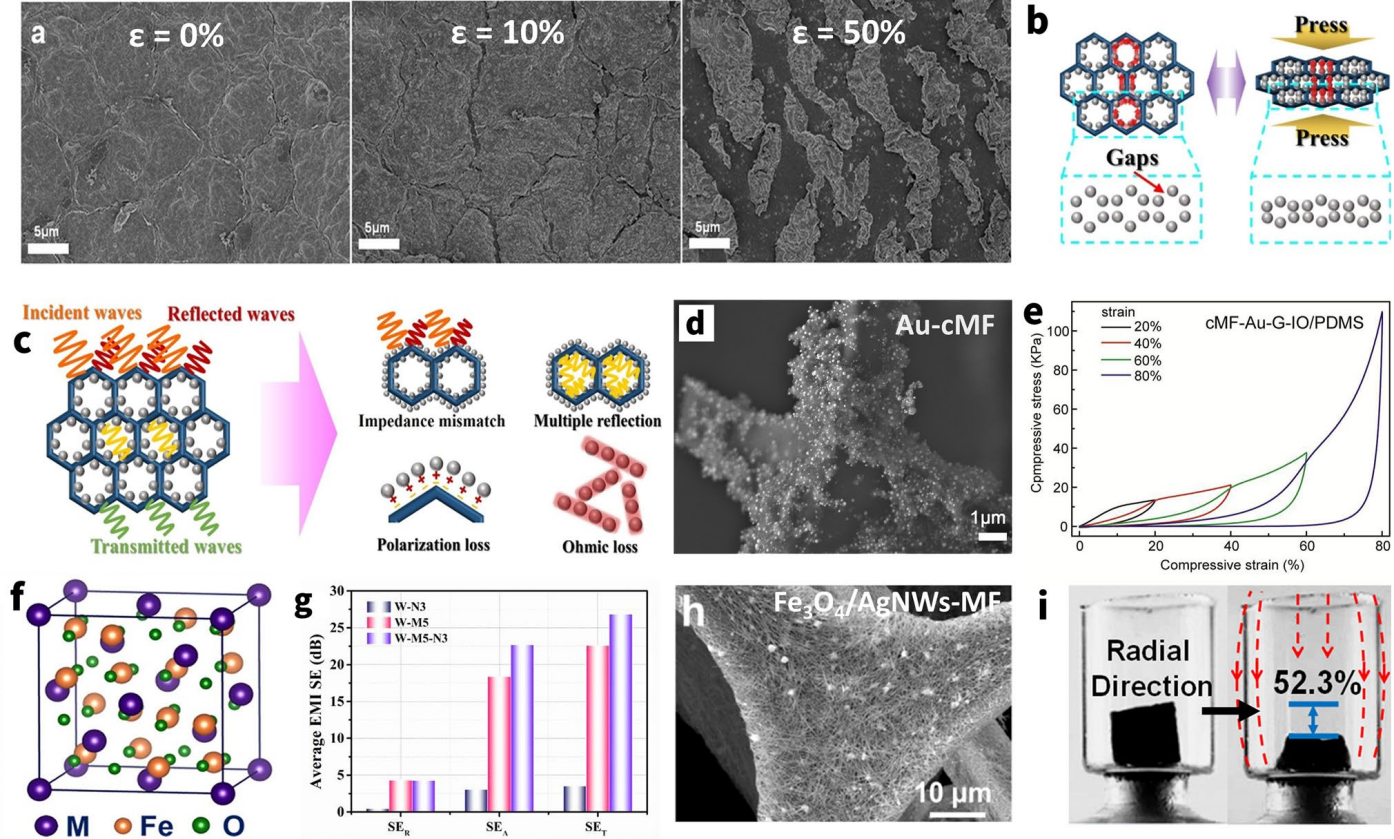
Nanoparticle based elastic EMI shielding composites. **a** SEM images of silver NPs/SBS composites before elongation and after elongation at strain of 10% and 50%. **b** Illustration for the change of nano/micro gaps in the compressive AgNPs sponge. **c** Schematic diagram of EM waves transmission in the AgNPs sponge. **d** The SEM images of AuNPs/carboned MF. **e** Stress–strain curves of carboned AuNPs/ MF-PDMS composite. **f** Schematic illustration of a unit cell structure of spinel MFe_2_O_4_. **g** X-band average EMI SE values of WPU-3wt% NiFeO_2_ (W-N3), WPU-5wt% MXene (W-M5), and WPU-5wt% MXene-3wt%NiFeO_2_ (W-M5-N3). **h** SEM images of AgNWs/Fe_3_O_4_/MF foam. **i** Magnetic field-induced compression along the radial direction of Fe_3_O_4_/graphene aerogel. **a** Reproduced with permission [63]. Copyright 2016, Royal Society of Chemistry.** b, c** Reproduced with permission [73]. Copyright 2020, Elsevier Ltd.** d, e** Reproduced with permission [81]. Copyright 2018, Elsevier Ltd. **f**Reproduced with permission [99]. Copyright 2021, Elsevier B.V.** g** Reproduced with permission [126]. Copyright 2021, American Chemical Society.** h** Reproduced with permission [91]. Copyright 2021, American Chemical Society.** i** Reproduced with permission [84]. Copyright 2015, American Chemical Society

Furthermore, in stretchable composites with metal nanoparticles, interfacial friction and mechanical interlocking between metal nanoparticles increase during stretching, hence increasing their mechanical properties [70]. The capacity of the elastomer's macromolecular movement is intact since metal nanoparticles are linked to its surface, meaning that the rigid nanofillers don't hinder the membrane from elongating when stretched. As a result, this loading method enhances the mechanical characteristics of the elastomer membrane without diminishing its stretchability [60]. Nevertheless, the metal nanoparticles on the surface of a highly elongated sample tend to decrease the electrical conductivity by crack formation when subjected to large stress. For instance, Kang et al. [63] fabricated the AgNPs/SBS porous composite by template methods. The results show that when the AgNPs content is 66.5 wt%, the conductive $\sigma$ and EMI SE of the composites are ~ 7800 S m^−1^ and over 45 dB, respectively, which are approximately 42 and 33% less than those of the composites under stretching up to 100 times with 60% strain (Fig. 5a), whereas, as for compressive sponge/foam/aerogel, the existent isolated nanoparticles coating on the sponge hole walls prevent the efficient carrier movement. Upon compression, these gaps are sharply reduced, and thereby the connective percolation improves, leading to lower contact resistance and better electrical conductivity for composites (Fig. 5b) [65, 66]. Gu et al. [58] prepared the PU/PDA/AgNPs composites based on the AgNPs coated on the surface of PU sponge by the *in situ* reduction. The results show that the composite has a high EMI SE (~ 84 dB) with the help of AgNPs (Fig. 5c). As such, it is discovered that as the conductivity of composites increases ~ 100% at strain of 80%, their capacity to effectively shield EM fields would change as well [71].

Apart from as the predominant conductive fillers, hybrid fillers combining the CB [72], CNTs [62, 73], graphene [74, 75], and MXene [76] have also been reported for the formation of the elastic EMI shielding composites. Clear evidence proves that inserting the metal nanoparticles into the hosting conductive system diminishes the pristine resistance via providing extra conductive routes, thereby yielding a greater conduction loss for better shielding properties [77]. Liao et al. [61] introduced Au nanoparticles into the carbonized melamine foam (cMF) carrying systematic structural modifications with graphene, Fe_3_O_4_ and PDMS to obtain a specifically engineered EMI shielding composite (Fig. 5d). Due to the improvement of system by Au nanoparticles, the cMF-Au-graphene-Fe_3_O_4_/PDMS composite exhibited excellent electrical conductivity (81.3 S m^−1^) and distinguished EMI SE (30.5 dB) at the thickness of 2 mm. Simultaneously, in contrast to composites containing merely conductive fillers, the enhancement in the elastic characteristics of hybrid nanocomposites was accomplished by the high load transfer efficiency of metallic nanostructures in the other matrix, hence improving the tenacity of elastic behavior (Fig. 5e) [74]. For example, AgNPs/CNTs with SBE elastomer have been combined synergistically to prepare the highly compressible conductive composite foam. Compared with pure CNTs/SBS foam, the addition of AgNPs can form double efficient conductive paths, thus greatly improving the electrical conductivity as well as EMI shielding performance of the composite foam. Moreover, after deposition of AgNPs on the CNTs/SBS foam, it is worth noting that the compressive modulus and compressive strength of the composite foam have increased significantly at the strain of 50% [73]. In another case, Gong et al. [62] reported an Au@CNTs/sodium alginate/PDMS flexible composites with high flexibility and good EMI performance. The EMI SE value of Au@CNTs/SA/PDMS composites with 1% content is 10 dB higher than that of CNTs/SA/PDMS composites with the same content, representing a significant improvement. Additionally, the composite materials basically go through elastic deformation when their elongation is less than 10%, which exhibits great flexibility.

##### Carbon

Graphite and carbon black (CB) were used as conductive fillers long ago to prepare EMI protection materials such as conductive rubber and wave-absorbing coatings [78–80]. In terms of loss mechanism, the loss of carbon particles is resistive loss type. When the macroscopic current caused by carriers increases, it promotes the conversion of EM energy into thermal energy, thus improving the EMI shielding performance. It also relies on the electron polarization, ion polarization, molecular polarization, and interfacial polarization attenuation of the medium to absorb EM waves. In practical applications, the incorporation of carbon particles into polymer elastomers, such as SBS [72] and silicone [80–82], allows the preparation of stretchable, high-performance conductive elastomers that shares the inherent advantages with silicone, including excellent thermal stability and climate resistance. Currently, the majority of elastomers use carbon black as an extra conductive material for EMI shielding system construction. In one case, Sun’s group prepared the CB-Ag@SBS hybrid foam by templates assisted fabrication using the CB and AgNPs as the conductive filler, SBS as the polymer matrix [72]. The result indicated that with the CB fraction of 15 wt% and a silver fraction of 0.63 vol%, the EMI SE of the CB-Ag@SBS hybrid foam reaches 81.3 dB at a thickness of about 5 mm. Simultaneously, adding the CB can improve the electrical stability at cyclic compression-release measurement of the foam. The results mentioned above present that the uniform distribution of carbon black in the SBS matrix can not only increase the interfacial stability of Ag nanoparticles with CB/SBS framework but also improve the mechanical–electrical stability of hybrid foams [83].

Furthermore, studies have shown that for carbon particles, which are traditional EMI shielding materials, surface treatment and hollowing treatments can take their performance to the next level. Zhao’s group firstly synthesized the conductive silicone rubbers composite filled with nickel-coated graphite (NCG) in order to boost the conductivity and EMI SE by coating the nickel [82]. In another study, Zhang’s group prepared the hollow carbon black (HCB)-based conductive rubber composites. The unique hollow morphology produced a better compression recovery of HCB than other solid carbon black, such as acetylene black [81]. Due to the hollow structure, the conductive silicone rubber composites were featured by high stretching resilience, fast compression recovery and excellent conductivity to satisfy the EMI shielding requirements.

As a 0D conductive filler, from the perspective of constructing a conductive network, the filler level is too high, and the corresponding expense will increase while the tensile stability decreases, according to the percolation theory. Therefore, 0D nanoparticles should act more as the secondary filler in EMI shielding films, used to enhance the shielding ability of conductive networks built of 1D or 2D nanofillers, rather than being used alone.

#### Magnetic Nanoparticles

##### Ferrites

Ferrites are typically ceramic materials of the ferrous group and one or more other appropriate metallic elements which, in terms of their electrical conductivity, are semiconductors, but are employed as magnetic media. Additionally, benefited by its distinctive crystal structure and excellent magnetic properties, spinel-type ferrite MeFe_2_O_4_ (M = Fe, Mn, Ni, Zn, Mg, etc.) stands out among them due to an extremely wide range of potential applications in the microwave domain (Fig. 5f) [84]. MeFe_2_O_4_ components may contribute to EMI shielding performance by virtue of their enhanced impedance matching and mild magnetic loss, resulting in greater EM wave dissipation [85–87].

Whereas, the conductivity of EMI shielding materials is intended to exceed the target value (1 S m^−1^) in commercial applications, hence the absence of a conductive filler renders a magnetic material ineffective for shielding [88]. Therefore, novel methods for enhancing the conductive property while maintaining magnetic loss are strongly preferred for effectively shielding EMI; this is seen as a desirable option. Yu and co-workers [89] exploited the NiFe_2_O_4_ to improve impedance and enhance magnetic attenuation of the MXene Ti_3_C_2_T_*x*_/waterborne polyurethane (WPU) composites and then developed a NiFe_2_O_4_-MXene/WPU hybrid aerogel through freeze-drying. The results show that the EMI SE of the NiFe_2_O_4_-MXene/WPU hybrid aerogel reaches 26 dB when the MXene and NiFe_2_O_4_ content are 5 and 3 wt%, respectively, which are largely higher than those of pure NiFe_2_O_4_ aerogel that is merely as low as about 5 dB. As illustrated in Fig. 5g, it is interesting to note that a sizable synergistic impact is observed because both SE_T_ and SE_A_ of hybrid aerogel are higher than the sum of their individual peers. Evidently, the magnetic–dielectric synergistic effect derives mostly from enhanced absorption as opposed to reflection [90]. Likewise, Zhao’s group prepared the Fe_3_O_4_–AgNW/melamine–formaldehyde foam by dip-coating method using Fe_3_O_4_ and AgNWs as fillers and MF foam as matrix (Fig. 5h) [91]. At a high-conductivity system, the SE_T_ values increase from 0.06 dB for the Fe_3_O_4_/MF components aerogel to 49.0 dB for Fe_3_O_4_–AgNWs MF aerogel with a thickness of 5 mm in the X-band. And the later aerogel demonstrated superior absorption-dominated EMI shielding ability with a particular EMI shielding effectiveness value of 4537 dB cm^2^ g^−1^. Moreover, in comparison with pure MF foam, the stress strain of composite foam took an upward trend with the load of Fe_3_O_4_, which could still complete the entire cyclic process, indicating outstanding elastic stability.

Some studies have focused on manipulating magnetic field-induced variations concerning about the distribution feature of 0D nanomagnets and the geometrical morphology of overall composites, both of which determine the EMI shielding behavior closely.

It is reasonable to believe that the gradient structure was beneficial to improve the impedance matching, which allow more EM waves to enter the composite material instead of being reflected, further improved the EM wave absorption efficiency of the composite material [92–94]. Simultaneously, it is straightforward and feasible to wirelessly control the gradient arrangement of magnetic particles. For example, Zhang and co-workers [95] employed the freeze-casting method to fabricate a hydrogel by filling Fe_3_O_4_ nanoparticles into poly(3,4-ethylenedioxythiophene)-poly(styrene sulfonic acid) (PEDOT:PSS) and polyvinyl alcohol (PVA) composite aqueous solution. Automatically, a gradient hierarchical structure is self-assembled with PVA under the effect of magnetic field force, and the induced dipole force may resist the sinking action of gravity, adding to the superior mechanical properties of the hydrogel. Consequently, Fe_3_O_4_ nanoparticles will absorb the energy and avoid local energy accumulation when the hydrogel is subjected to a large tension strain (> 100%), thereby enhancing the mechanical capabilities of hydrogel.

Additionally, the magnetic field-induced phenomena, apart from that of nanomagnets dispersion for the EMI shielding performance and elasticity of composites, include the deformation about geometrical morphology of overall composites as well [96–98]. Yury’s group [99] synthesized 3D graphene aerogels decorated with Fe_3_O_4_ nanoparticles by freeze-dried. The results show that the ultralight magnetic aerogels exhibit up to 52% reversible magnetic field-induced strain and strain-dependent electrical resistance, both of which could be utilized to monitor the degree of compression/stretching of the material (Fig. 5i). Available evidence indicates that the EMI SE may alter with the thickness, conductivity, and internal 3D porous architecture of aerogel under applied strain, and thus it might pave the way for the development of reconfigurable EMI shielding materials with the wireless control.

##### Transition Metal

Transition metals, including Fe, Co, and Ni, have an innate magnetic property that allows their particles to interact strongly with high-frequency EM waves and, in theory, effectively lose EM waves [77, 100–104]. It is intriguing seeing as interacting with EM waves, the transition metal particles exhibit characteristics that fall midway between those of the previously discussed ferrites and highly conductive nanoparticles. In contrast to ferrite, ferromagnetic metal particles have a very straightforward crystal structure. Consequently, there is no magnetic moment extinction across magnetic sublattices in these particles, as there is in ferrite. Therefore, the magnetic characteristics of transition metal particles are stronger than those of ferrite, and their saturation magnetization strength is typically greater than four times that of ferrite, which can result in exceptionally significant magnetic loss [105]. Due to the confinement effect and the tiny size effect of the nanoparticles, the ferromagnetic resonance was primarily responsible for the magnetic loss of the transition metals nanoparticles, where natural resonance acted at low frequencies (< 10 GHz) and exchange resonance functioned at high frequencies (> 10 GHz) [106].

When the Ni particles were included in the composites, the values of *ε*' and *ε*″ shot up significantly, indicating an increased capacity for dielectric loss [107]. This is primarily attributable to the elevation in conductivity that has taken place. While the conductivity of nickel particles isn't quite up to the level of silver's, they can nevertheless increase the conductivity of the composite as a metal, particularly when compared to ferrite. Furthermore, such an acceptable conductivity was helpful in reducing the impedance mismatch at the air-composite contact interface. It enabled more EM waves to enter the sponge and subsequently be absorbed within the sponge, resulting in lowering the reflection loss. The remnant EM waves will be partially absorbed and dissipated as a result of magnetic hysteresis loss and eddy current loss [108, 109]. As with ferrite, however, magnetic loss alone is insufficient for effective EMI shielding. The inclusion of nickel particles is like the cherry on top for EMI shielding of material. Wu and co-workers synthesized the decorated polyester/Fe_3_O_4_ textile composites by an *in situ* formation of Fe_3_O_4_ and then obtain the Ni@decorated polyester/Fe_3_O_4_ composites by electroless deposition of Ni on a PET fabric. The result shows that Ni@decorated polyester/Fe_3_O_4_ exhibited a moderate EMI SE (13.4 dB), while being much more than that of Ni@decorated polyester/Fe_3_O_4_ (0.02 dB). Unfortunately, it still falls short of EMI shielding requirements. To achieve good EMI shielding performances, it was required to strike a compromise between the electrical and magnetic properties of the composite sponge [110]. For instance, Wang’s group prepared the Ni/Polypyrrole (PPy)/Polyethylene terephthalate (PET) fabrics by in situ polymerization and subsequent electroless plating of nickel. The Ni particles is uniformly distributed on the PPy/PET fibers, thus constructing the heterogeneous structure automatically [101]. With the help of this coaxial structure, multiple reflections at interfaces can be efficiently facilitated, and the electrical and magnetic properties for EM attenuation may be greatly integrated. The results show that when the nickel-plating time and the in situ polymerization time are both 2 h, the EMI SE of the Ni/PPy/Non-woven PET fabric and the Ni/PPy/Warp knitted PET fabric are 77.87 and 62.60 dB at the X-band regime, respectively. Besides, due to the nature of the metal and the deposition process, Ni layers have a very restricted elongation at break and are invariably broken before polyester layers.

There are two development avenues for the use of transition metal particles in elastic EMI shielding films. The first involves enhancing the inherent morphology of the particles, shrinking the size of the particles, and creating high-aspect-ratio transition metal nanowires [24]. The unique effect of nanoparticles and the anisotropy of nanowires can be leveraged to improve EMI shielding performance. The second involves changing the particle system to form a core–shell structure out of materials with increased conductivity, promoting interfacial polarization and numerous reflections at the interface [45].

### 1D Material

#### 1D Nanocarbon

In recent years, a fresh upswing in EM wave shielding has recently been brought about by the introduction of 1D nanocarbons. Additionally, their exceptional chirality and electric characteristics indicate exceptional potential for EM wave shielding and absorption. 1D nanocarbons may be divided into CNTs, carbon nanofibers, and carbon nanocoils based on structural distinctions, of which CNTs are by far the most popular and thus will be highlighted below.

CNTs with large aspect ratios have been performed to ameliorate the EMI SE of the material as one of the most common fillers, which showed lightweight, excellent mechanical properties, electrical conductivity, good thermal conductivity and low-cost [111, 112]. Among the most attractive advantages of these materials is their ultrahigh anisotropy ratio for increasing dielectric loss capacity. Meanwhile, it is widely accepted that an ideal shield must block all the EM waves by the means of absorption. In contrast to metals, proper conductivity may lessen the impedance mismatch between nanocarbons and the incident space of EM waves, enabling greater absorption rather than reflection of EM waves [113]. When compared to other nanocarbons, CNTs with high aspect ratios need less fractional volume to obtain equivalent conductivities, and hence their composites often exhibit higher elastic characteristics.

**Fig. 6:**
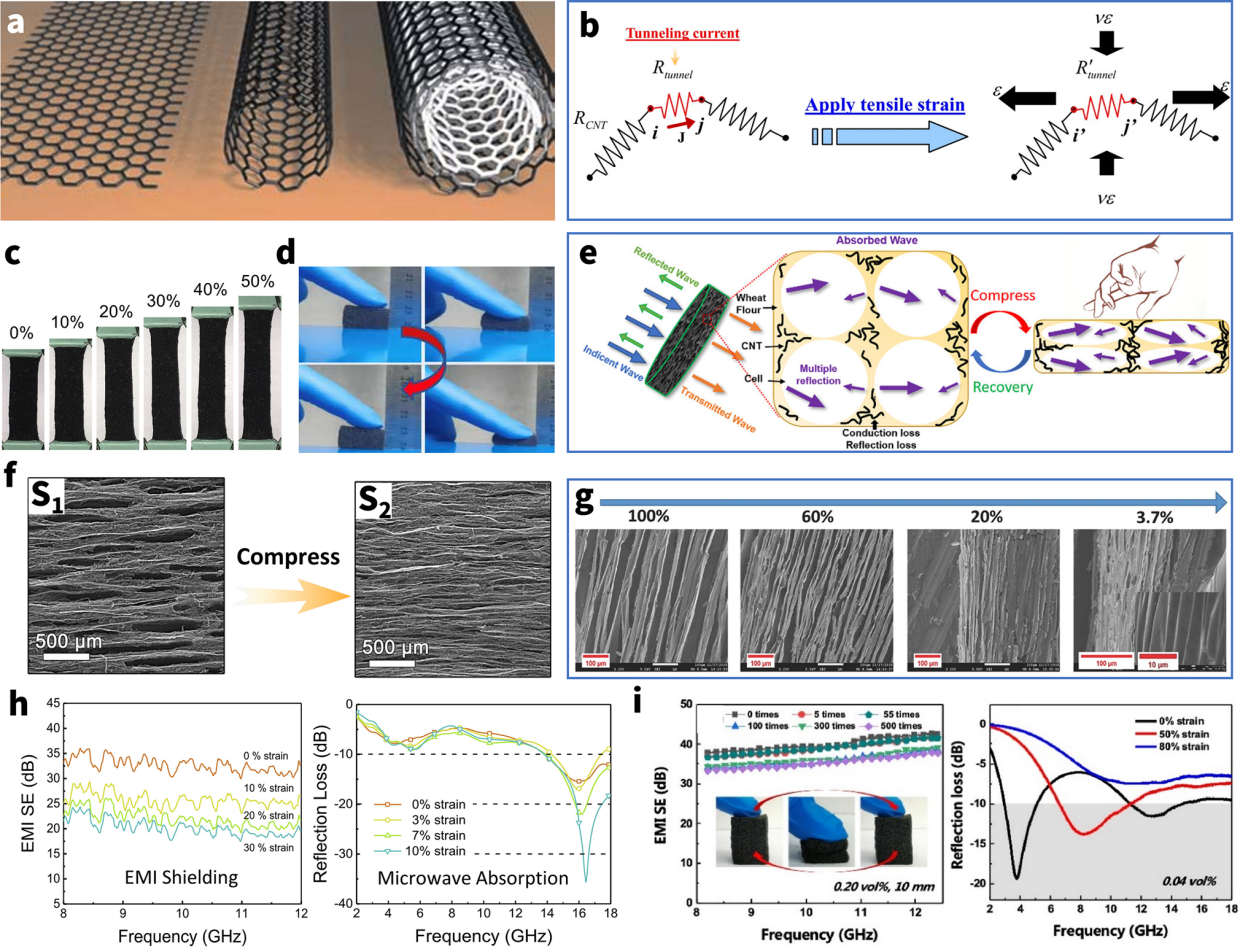
**a** Basic forms of planar graphene sheet, SWCNT, and MWCNT (from left to right). **b** Modeling of tunneling effect among neighboring CNTs applied tensile strain. **c** Digital images of CNTs/PU-Ecoflex composite foam under the stretching strain of 0–50%. **d** Digital images of the excellent compressibility of CNTs/PIF-PDMS composite foam. **e** Schematic illustration of EMI shielding mechanism for compressible foam. **f** SEM images of CNTs-Wood aerogel in the pristine state (S_1_) and compressed state (S_2_). **g** SEM images of microstructures of porous MWCNT/WPU composites at various percentages of original thickness. **h** EMI SE (Left) and RL (Right) of a CNTs/PU-Ecoflex composite foam under various stretching strains.** i** Left: SE curves of CNTs/ PU-TPI foam (~ 0.20 vol% CNTs) during multiple compressive deformation and recovery cycles. Right: RL curves of CNTs/ PU-TPI foam (~ 10 mm in thickness) under different compressive strains. **a** Reproduced with permission [305]. Copyright 2005, Springer Nature.** b** Reproduced under the terms of the CC-BY Creative Commons Attribution 2.0 Generic license (https://creativecommons.org/licenses/by/2.0) [128]. Copyright 2012, The Authors, published by Springer Nature.** c, h** Reproduced with permission [123]. Copyright 2019, Elsevier Ltd.** d** Reproduced with permission [285]. Copyright 2022, Tsinghua University Press.** e** Reproduced with permission [262]. Copyright 2021, American Chemical Society. f Reproduced with permission [21]. Copyright 2021, Elsevier Inc.** g** Reproduced with permission [111]. Copyright 2017, Wiley-VCH.** i** Reproduced with permission [125]. Copyright 2022, Elsevier Ltd

According to the number of concentric graphene cylinders, CNTs could be divided into two types, including single-walled carbon nanotubes (SWCNTs) and multiwalled carbon nanotubes (MWCNTs). While single-walled carbon nanotubes (SWCNTs) are the simplest kind of nanotube and are made by curling sheets of graphite, multiwalled carbon nanotubes (MWCNTs) are generated when carbon tubes of varying diameters stack up in a multilayer structure due to van der Waals interactions (Fig. 6a) [114]. Theoretically, the current carried by CNTs should be substantially greater than that of traditional metal wires owing to the ballistic transport characteristics of electrons in CNTs. Due to the preparation process's limitations and flaws, it was not, however, achieved. Certain experimental findings show that the overall conductivity of single-walled carbon nanotube networks (~ 17 × 10^7^–2 × 10^7^ S m^−1^) is much higher than that of MWCNTs (~ 5 × 10^3^–5 × 10^6^ S m^−1^) due to the difference in intrinsic resistance [115, 116]. Likewise, when MWCNTs and SWCNTs are combined with polymers to create composites, the mechanical strength and strain-to-failure (*ε*_b_) of the resulting materials vary depending on the CNT composition [117]. Consequently, both and their composites exhibit distinct variations in EMI shielding characteristics and deformability.

Moreover, the conductivity of the CNTs percolation network drifts under strain conditions, which in turn causes a change in EMI SE [118]. Hu et al. [119] first theoretically demonstrated that the contribution of the piezoresistivity of CNTs on the total piezoresistivity of the nanocomposite is comparatively small, compared with those from the change of the internal conductive network and tunneling effects in junctions (Fig. 6b). It further reveals that CNT-based elastic EMI shielding materials change shielding performance due to deformation of the entire percolation network and changes in tunneling distances between CNTs [120]. In particular, for well-developed percolation networks of highly concentrated composites, increasing applied strain results in network deformation and displacement, but does not result in a discernible SE reduction since there are still plenty of conductive routes available. Therefore, effective EMI shielding exists while there are still a sufficient number of nanotube interconnections. Lu et al. [121] successively prepared a flexible spongy CNTs consisting of self-assembled, interconnected CNT skeletons, with a density of 10.0 mg cm^−3^, which directly used as EMI shielding film. The freestanding CNTs sponge with a thickness of 1.8 mm shows highly EMI SE and SSE of 54.8 dB and 5480 dB cm^3^ g^−1^ in X-band, respectively. It is noteworthy that the composite still maintains its high SE performance and structural integrity even after 1000 cycles of stretching tests. However, subsequent transition to poor shielding occurs when disconnection of conductive fillers after highly stretching cycles becomes unavoidable. Feng et al. [122] fabricated segregated CNTs/PU composites by the intense selective sintering methods. Besides, they experimentally demonstrated that the EMI SE of CNTs/PU composites decreases from ~ 35 dB (2.00 mm) at pristine state to ~ 12 dB (0.91 mm) at 200% tensile strain. Likewise, Huang et al. [123] fabricated the CNTs/PU foam with hierarchical buckling structure and then filled with Ecoflex by vacuum infiltration so as to obtain a stretchable EMI shielding materials (Fig. 6c). It is gratifying to note that the EMI SE of CNTs/PU-Ecoflex composites can still reach 20 dB under 30% stretching strain. Generally, these drastic changes can be attributed to the reduction of material thickness due to stretching and the physical disconnection of some of the CNTs. Meanwhile, inherent exponential drop of the EMI SE is seen to be determined by the tunneling mechanism of CNT/polymer nanocomposites.

In addition to acting as a filler for pulling stretchable EMI shielding films, carbon nanotubes are also used to construct lightweight, efficient, and stable compressible porous EMI shielding materials [124]. Recently, Sun et al. [125] introduced CNTs to carbon skeletons derived from the isocyanate-based aromatic polyimide foams (PIFs) so as to fabricate the high-performance CNTs/PIF-PDMS composite foams for EMI shielding. The result shows that the EMI SE decreased from the original value of ~ 57.6 to ~ 54.6 dB at 30% strain after the first compression, followed by ~ 48 dB (at 50% strain) and ~ 40 dB (at 80% strain). This shows that the foam can still adequately meet the EMI shielding requirements even at a high compression of 80%, further demonstrating the stability of the conductive network formed by the CNTs. Meanwhile, the cyclic compression test also shows that the foam has excellent compression resilience, which can withstand repeated deformation of flexible electronic devices while maintaining the morphological function intact (Fig. 6d).

Furthermore, compared with traditional non-deformable materials, such as dense CNT films, CNT compressible foams can be explored to probe the connection between key factors such as material thickness and electrical conductivity with EMI shielding performance by simply applying different strains to the same material. This not only facilitates the elucidation of the endogenous mechanism of the variation of EMI shielding performance of compressible shielding materials, but also helps to explore the potential relationships between the influencing factors, further contributed to the design of other non-compressible EMI shielding materials.

According to Eqs. 3 and 4, the EM wave loss resulting from EMI shielding of the material is roughly related to the material thickness and conductivity, which should logically apply to CNT foam.

(i) Thickness

Chen et al. [126] uniformly mixed CNTs and wheat flour (WF) in a surfactant solution to form a stable sol system, and then heat to transform from sol to gel, which was directly freeze-dried to obtain the CNT/WF aerogels with a homogeneous porous structure. The average SE_total_ value of the CNT/WF (3%) foam with a thickness of 5 mm was about 40.1 dB, which far exceeds the requirements for the practical use of EMI shielding materials. The CNTs wrapped around the gluten protein backbones made touch with one another when the force was applied to the WF/CNT foam. As a consequence, the distance between the CNTs was greatly decreased, increasing the CNTs' contact area and decreasing the electrical resistance. Although the electrical conductivity increases with the compact CNT contact during the compression deformation, the decreasing thickness gave less opportunities for the interference of the incoming waves with the CNT and cell walls, resulting in the poor EMI shielding of the CNT/WF foam (Fig. 6e). From the Eq. 3, it is obvious that the thickness weighs more heavily in the SE_A_ than the conductivity-related impact.

(ii) Conductivity & complex permittivity.

The conductivity also becomes the most important component in determining the EMI shielding performance when it varies dramatically and exponentially in comparison with the thickness. Liu et al. [21] used acidulated-CNTs as the conductive filler, wood sponge as the matrix to prepare the wood/CNT sponge composites via dip-coating. Due to the special pore structure inside the wood sponge, it will make the carbon nanotubes adhere to the pore surface, thus losing contact and not forming a complete and stable conductive pathway (Fig. 6f). This insufficient conductive network will make the overall electrical conductivity of the sponge drop significantly. And the material conductivity only will be significantly increased when compressed because the pore walls are in contact with each other. This steep change in conductivity can also lead to a dramatic change in EMI SE, from wave-transparent to perfectly shielded. Meanwhile, the change in conductivity during compression also affects the complicated permittivity of CNT composites. Moreover, Wang et al. [127] synthesized the CNT/PU foams and test them with the increasing of compressive strain. This behavior leads to more physical contacts between cell skeletons and benefits to the formation of more horizontal conductive path perpendicular to the incident direction of EM waves, further yielding a rise in complex permittivity during compression process. And this may increase the SE_R_ due to the enhancement of *ε*’.

(iii) Interior structure

The inner pore structure variation of the material also impacts the EMI loss power, including multiple reflections, in addition to the material's macroscale electrical and geometric properties. Zeng et al. [128] focused on the intrinsic shielding mechanism of the porous materials. They demonstrated that the compression reduces the pores and in in turn the multiple reflections by an in situ compression experiment (Fig. 6g). Besides, in the porous CNT-materials, the absorption behavior dominates the total shielding, whereas in the final-state dense CNTs films, the significant contribution from the reflection makes SE_T_ even higher than that of the porous material at the low CNT mass ratios because of deteriorative impedance mismatch.

Notably, in composites with low concentrations of carbon nanotubes (less than ~ 10 wt%), the conductivity is relatively low and may only meet the minimum requirements for EMI shielding, if at all [31]. But the material may potentially be converted into a superior microwave-absorbing material when combined with the ideal reflective layer needed for such materials [129]. Huang et al. [123] introduced different volume concentrations of CNTs into swelled PU foam and subsequently filled with Ecoflex by vacuum infiltration to obtain EMI shielding composite foams (~ 4.3 vol% CNTs) and microwave-absorbing composite foams (~ 0.43 vol% CNTs), respectively. With the stretching of the material, the EMI SE of shielding composites has a certain decline, while the minimum reflection loss (RL) (i.e., the peak absorption point) of the microwave absorption composites occurs in the direction of low-frequency shift, due to changes in thickness and structure (Fig. 6h). Likewise, Wang et al. [127] branched different concentrations of CNTs and trans-1,4-polyisoprene (TPI) to endow the PU frameworks as EMI shielding foams (~ 0.2 vol% CNTs) and microwave absorbent (~ 0.04 vol% CNTs) to protect against EM wave disorder interference using the template method. The experiments showed that the foam for EMI shielding was resilient and could repeat 500 compression-recovery tests while maintaining the performance, while the absorption material also produced peak shifts (Fig. 6i). For the dynamic conductivity of CNTs, it is possible to use the same functional material to prepare two EM protection materials with different functions. At the same time, the preparation just needs to modify the introduced concentration, which simplifies the process stages and encourages large-scale scalable manufacturing.

#### Noble Metal Nanowires

Despite the widespread use of nanocarbon as a conductive filler, the material's subpar EMI SE performance remains a significant barrier to widespread adoption in shielding applications. As an alternative, noble metal-based nanowires are becoming effective shielding fillers because of their high electrical conductivity by nature [130]. Particularly, illustrative metal nanowires including gold nanowires (AuNWs) [131–135], AgNWs [136–140], copper nanowires (CuNWs) [141] are utilized in a range of stretchy conductive materials due to their ultrahigh anisotropy ratio, which facilitates the formation of a more stable conductive network. In addition, the composites made from nanowires with high aspect ratios tend to exhibit superior elastic characteristics because these nanostructures require smaller fractional volumes to obtain the same conductivities as conventional 0D or 2D nanomaterials [57]. Nonetheless, the frequency of scientific research and industrial applications of each nanowire in elastic EMI shielding films varies greatly in the view of the difficulty of preparing noble metal nanowires, the cost of preparation, and the properties of the corresponding bulk metal [18]. This section gives a thorough explanation of AgNWs and CuNWs for elastic EMI shielding materials depending on their application range and performance.

##### AgNWs

**Fig. 7:**
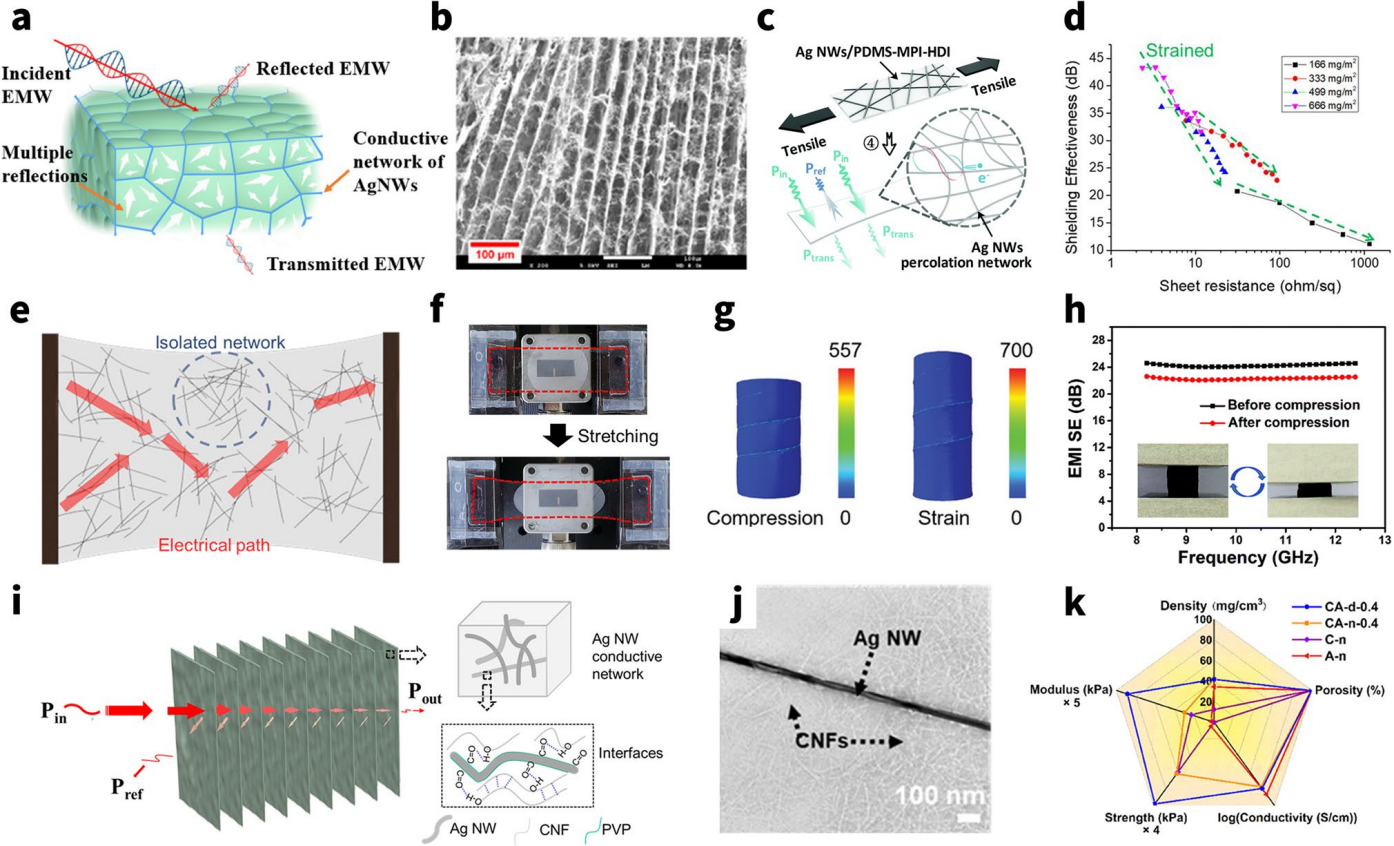
AgNWs-based EMI shielding composites. **a** Schematic illustration of shielding mechanism of AgNWs composites. **b** SEM image of microstructures of the AgNWs-PVP aerogels in longitudinal planes (scale bars are 100 μm). **c** Schematic diagram of the stretched AgNWs composite against EMI. **d** EMI SE change plotted against sheet resistance. **e** Schematic illustration of proposed description for up-shifted shielding effectiveness during stretching. **f** Digital images of EMI shielding test under stretching condition. **g** Finite element analysis (FEA) of AgNWs sponge stress condition during single skeleton was being compressed, and strained. Color bar: MPa. **h** EMI SE of the AgNWs/MXene hybrid sponge before and after the 500-cycle fatigue test with a compressive strain of 60%. **i** Schematic showing the proposed EMI shielding mechanism of the lamellar porous AgNWs/CNF aerogels. **j** TEM image showing the good attraction between the CNFs and AgNWs and adhesion of CNFs on the AgNWs. **k** Comparison of several characteristics for CNF, AgNWs, and the composite sponges. **a** Reproduced with permission [306]. Copyright 2022, American Chemical Society.** b** Reproduced with permission [29]. Copyright 2020, American Chemical Society.** c** Reproduced with permission [144]. Copyright 2021, The Royal Society of Chemistry.** d**-f Reproduced with permission [36]. Copyright 2022, American Chemical Society.** g** Reproduced with permission [148]. Copyright 2019, Wiley–VCH.** h** Reproduced with permission [287]. Copyright 2021, The Royal Society of Chemistry.** i**-**j** Reproduced with permission [30]. Copyright 2020, American Chemical Society.** k** Reproduced with permission [142]. Copyright 2020, American Chemical Society

AgNWs are considered the most promising noble metal nanowires owing to the highest bulk electrical conductivity (6.3 × 10^7^ S m^−1^) and exceptional air-stability, making it commonly used in EMI shielding field. These AgNWs, once embedded as the EMI shielding materials, would form mesh-like percolation network structures that facilitate the free movements of carriers through contact junctions and the filler materials, even when mechanically deformed by applied strains. Since electrical conductivities can be increased generally, this stable structure may cause a considerable impedance mismatch with the space medium incident on the EM wave. Therefore, the majority of the EM waves are reflected at the interface due to this impedance mismatch, which is directly caused by the free electrons that have accumulated on the surface of the extremely conductive network for AgNWs (Fig. 7a). For example, Zeng et al. [29] constructed the AgNWs percolation network in the form of aerogel using the unidirectional freeze-drying process. The AgNW aerogel with 2.3 mm thickness exhibits the conductivity of approximately 1400 S m^−1^ and the maximum EMI shielding performance of 72.5 dB at a density of 27.6 mg cm^−3^.

Additionally, the highest electronic conductivity and high anisotropy ratio of AgNWs can both be used to modify the dielectric permittivity of hybrid materials. For instance, Li et al. [142] compared the complex permittivity of composites before and after the introduction of AgNWs. They discovered that AgNWs can increase the dielectric loss due to both the conductivity and interface of composites, which will primarily endow the composites with superior EMI shielding ability.

It is highly challenging to create a stable self-supporting structure for AgNWs-based EMI shielding elastomers using only AgNWs, and even when it is possible, such as aerogel (Fig. 7b), it still has a low mechanical strength at low densities, which limits its application [29]. Typically, one of the widely used methods to produce high-performance shielding elastomers is to combine silver wire with aided polymer to create a high-quality composite. The efficient infiltration of assisted polymer could not only support the whole architectures but also enhance the mechanical properties of composites (e.g., Young’s moduli and tensile strengths), which plays a key role in protecting the percolated AgNWs network by dissipating the applied strain [57]. Depending on the loading conditions, common assisted polymers are mainly divided in two categories: one is the stretchable polymers and the other is compressive polymers.

The matrix polymer of stretchable assistance materials, including PDMS, PU, etc., possess exceptional mechanical toughness and intrinsic deformability. When the stretchable polymers are added to the mix, they help to constantly stabilize the morphology of the AgNWs network and improve the mechanical stability of the overall composites. Li and co-workers compared the configurations of AgNWs and AgNWs/PU and proposed that nonaffine deformations (reorientation and buckling) of AgNWs are greatly reduced by the mechanical constraint from the PU layer [143]. And polymer can also bestow the composites with good stretchability and EMI shielding effectiveness beyond just stabilizing the AgNWs network. Sun et al. [144] first presented the transparent, stretchable and self-healable EMI shielding materials by taking designed PDMS-based silicone elastomer as a substrate for embedding AgNWs. The EMI shielding performance gradually decreases from ~ 32 dB at pristine state to ~ 22 dB at 50% tensile strain because of the destruction of the conductive network caused by stretching (Fig. 7c). Moreover, Jung et al. [36] first reported a highly stretchable EMI shielding layer with silver nanowire percolation network on elastic PDMS-based substrate and then test its tensile performance (Fig. 7d–f). They noted that for the percolation network with dense distribution of AgNWs, i.e., a surface density of 666 mg m^−2^, the EMI SE is maintained at 20 dB or larger even at a large strain of 50%.

Meanwhile, sustaining EMI SE after numerous reduplicated stretching–retracting cycles is very vital for stretchable EMI shielding materials to effectively shelter the next-generation wearable electronics from EMI. Appropriately, the polymer elastic matrix can create the robust interfacial adhesion with the AgNWs framework, which makes these composites very mechanically/electrically stable. Jia et al. [145] integrated AgNWs and conformal PU layers on a carbon fiber fabric in order to fabricate a highly electrically conductive fabric with the ultrahigh EMI shielding performance. Due to the good mechanical deformability of PU, it was worth noting that conductive fabric with seven dip-coating cycles maintained a superior EMI SE of 87.7 dB even after 100 stretching–retracting cycles, indicating 83% retention of the original EMI SE.

Considering assisted polymers for compressive AgNWs networks, these polymers, such melamine, cellulose, and other forms of polymer, have the capacity to support AgNWs in the formation of stable composites with good strain recoverable compressibility and fatigue resistance [43]. Certainly, there should be no doubt that EMI shielding performance is guaranteed even after compressive deformation at any strain. Due to the unique morphology of silver nanowires, it is more suitable for wrapping and winding on the surface of other polymeric compressed elastomers, known as the self-locking structure (Fig. 7g), to act as an EMI protection network rather than forming a stand-alone film. Recently, Wang et al. [146] report on lightweight MXene/AgNWs/melamine hybrid sponges featuring porous structures that are fabricated by dip-coating method (Fig. 7h). Benefiting from the support of matrix, the sponges exhibit a large recoverable compression strain (80%), and fatigue resistance. The average EMI SE of the hybrid sponge only decreases from 24.3 to 22.3 dB, exhibiting a high retention of 91.8%, after the 500-cycle fatigue test with a compressive of 60%. In another study, Lin et al. [147] revealed that the pressure to AgNWs is higher than that of melamine sponge skeleton because of the self-locking structure of AgNWs, and indicate that during loading process, the hybrid sponge acts as coil spring to assist sponge skeleton structure rebound (Fig. 7g). This silver coil spring of this design may distribute force evenly across a pliable base, halting any creep deformation that could otherwise occur. Additionally, this self-locking structure is benefit to maintain the SE_R_ during compressing intensely. Reversely, the excess strain render cracks in a commercial EMI shielding sponge (Ni-coated), thus causing dramatically decrease of SE_R_.

Contrary to elastic polymers, the significant hydrogen-bonding interactions between PVP on the AgNWs and the CNFs help to bind the cellulose nanofibers (CNFs) that are employed to help build the AgNW network (Fig. 7i, j). These strong interactions contribute to the successful assembly of the ultralight yet robust AgNW-embedded biopolymer aerogels. And the compressive strength and moduli display an initial increasing behavior due to the more effective interfaces between the AgNWs and CNFs [30]. Greiner and co-workers reported that a wood-inspired composite sponges consisting of CNFs and high-aspect-ratio AgNWs were generated with anisotropic properties by the directional freeze-drying [148]. It is worth mentioning that the sponge with 0.4 vol% AgNWs could exhibit a high EMI SE over 80 dB at X-band regime. Simultaneously, compared with the pristine brown–gray AgNW-only aerogel, the introduction of CNFs can enhance the physical and chemical interactions to form an effective continuous structure among AgNWs, avoiding collapsed with a very little force (Fig. 7k). Furthermore, the investigation conducted by Zeng and co-workers indicated that increasing AgNWs content in the porous nanocomposites may cause aggregation of the nanofillers, resulting in more stress concentration zones in the porous structure erected by AgNWs; consequently, the aerogels collapse more easily under the external compressive load [143, 149].

**Fig. 8:**
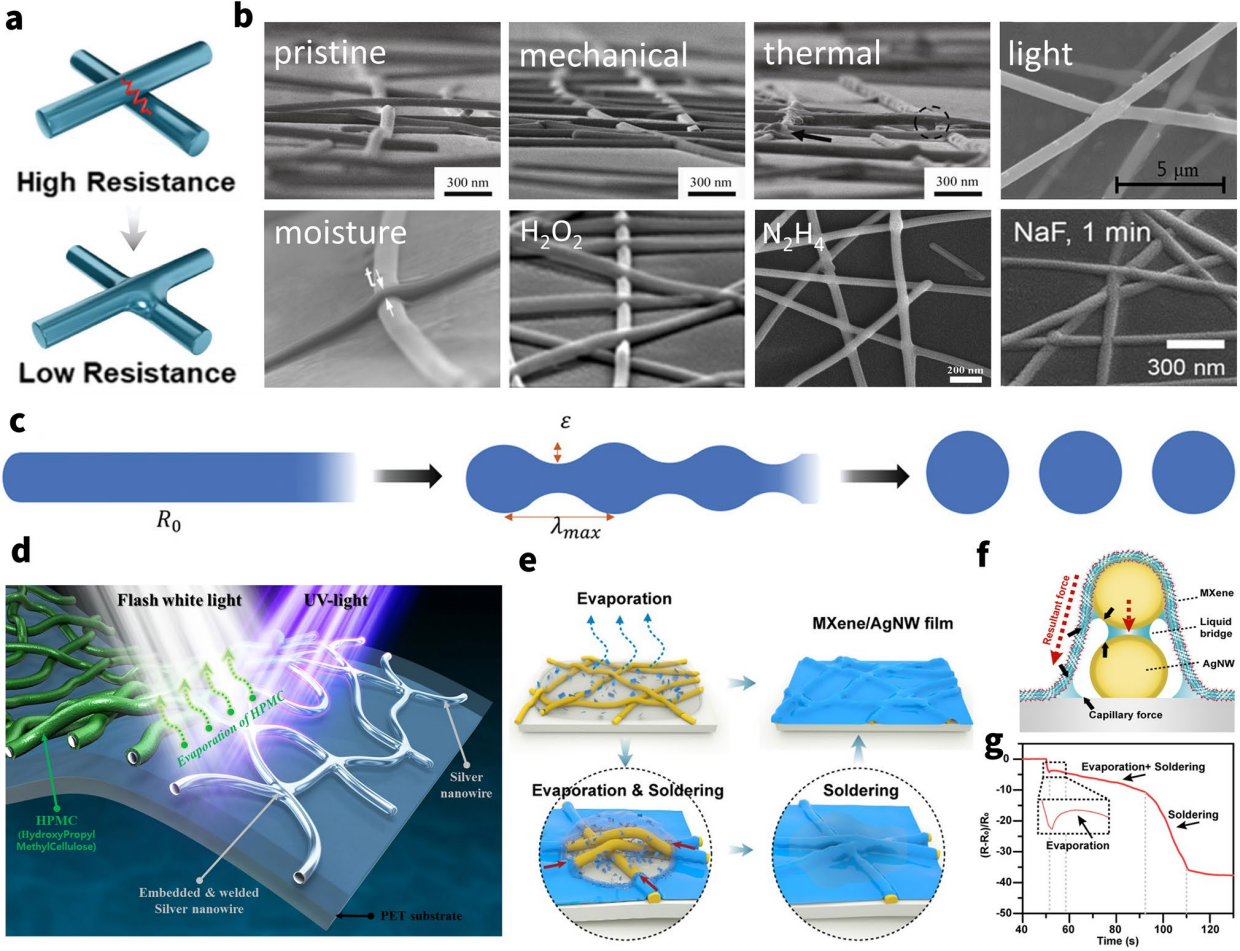
Welding techniques for AgNWs percolation network. **a** Schematic illustration of the welding technique. Reproduced with permission [131]. Copyright 2019, Royal Society of Chemistry. b SEM images of AgNW junctions before and after various welding methods. Image for “pristine”, “mechanical”, “thermal”: Reproduced with permission [156]. Copyright 2011, Tsinghua University Press and Springer-Verlag Berlin Heidelberg. Image for “light”: Reproduced under the terms of the CC-BY Creative Commons Attribution 4.0 International license (https://creativecommons.org/licenses/by/4.0) [159]. Image for “moisture”: Reproduced with permission [165]. Copyright 2017, American Chemical Society. Image for “H2O2”: Reproduced with permission [166]. Copyright 2016, American Chemical Society. Image for “N2H4”: Reproduced with permission [161]. Copyright 2019, Elsevier Ltd. Image for “NaF”: Reproduced with permission [162]. Copyright 2017, American Chemical Society.** c** Schematic of the progression of spheroidization of a long cylinder. Perturbations with a wavelength of approximately λmax tend to evolve fastest and cause the formation of spheres, indicating failure of the nanowire. Reproduced with permission [160]. Copyright 2020, Wiley-VCH GmbH.** d** Schematic diagram for combined flash light welding process of silver nanowire with HPMC binder. Reproduced under the terms of the CC-BY Creative Commons Attribution 4.0 International license (https://creativecommons.org/licenses/by/4.0) [159].** e** Schematic illustrating the evaporation & soldering procedure of a transparent MXene/AgNWs film.** f** Soldering mechanism of the MXene/AgNWs film.** g** Resistance changes of the AgNWs film in the soldering process.** e**–**g** Reproduced with permission [150]. Copyright 2020, American Chemical Society

Notably, a post-treatment of the already constructed AgNWs conductive network can significantly increase the overall reflection loss in addition to the strengthening of polymer on the elastic EMI shielding material. The electrical percolation of a AgNWs network depends strongly on the effective point contact at nanowire–nanowire [150]. However, many as-prepared nanowire films suffer from high contact resistance due to the nanogaps or weak contact at the junctions. This large contact resistance between nanowires would limit the conductivity of AgNWs network and slash the SE_R_, SE_A_ inevitably inhibiting their application in EMI shielding. Meanwhile, poor wire–wire contact also affects the mechanical deformability of the AgNWs elastic composites because the loosely stacked nanowires would easily move under deformation, leading to deteriorated conductivity [130]. The conventional but effective method to reduce the high junction resistance of AgNWs is welding (Fig. 8a) [151]. A variety of post-treatment techniques have been developed with the assistance of heating [152–154], light [155, 156], electricity [157], mechanical pressure [158], capillary force [159, 160] or chemical reagent [161] (Fig. 8b), while all have concerns in the application of flexible devices. Depending on the welding mechanism, these post-treatment techniques are mainly divided in two categories: One is the physical welding and the other is chemical welding.

Physical welding techniques could reduce the contact resistance by fusing the junctions of nanowires. When high thermal, mechanical, or optical energy is applied to a network of nanomaterials, they can be welded together, enabling facile electron transfer across the conductive filler network and then improve the shielding performance of the composites. For instance, Wong’s group fabricated a welded AgNWs aerogel through thermal treatment at 200 °C, further backfilled with PDMS to obtain AgNWs/PMDS elastomer [152]. Compared with pristine AgNWs/PDMS elastomer, the EMI SE sharply rises from ~ 20 to 35 dB after thermal welding. And shielding performance also exhibits good mechanical stability after 1000 stretching cycles derived from welded AgNWs skeleton and backfilling of an elastic polymer. Recently, Chen et al. [155] used rGO conformally wrapped AgNWs (AgNWs@rGO) as the conductive filler and PDMS as the substrate to prepare a AgNWs@rGO/PDMS transparent composite via selective electrodeposition and pulsed laser irradiation treatment, which can enhance the EMI SE and the stability during stretching of composites. It is noted that the one of the reasons about enhancement of EMI SE is both *ε’* and *ε’’* were obviously enhanced, ascribable to the improvement in the material’s electrical conductivity based on free electron theory and effective medium theory. In a nutshell, these welding techniques result in significantly reduced overall resistance and improved mechanical deformability. However, those techniques frequently have a lot of flaws [159]. For example, thermal heating requires an accurate control over the heating temperature and time to prevent spheroidization fracture of the metal nanowires (Fig. 8c) and damage to heat-sensitive substrates (e.g., PDMS, PU) [162, 163]; mechanical pressing may not be applied to some devices as the high pressure (up to 80 GPa) may destroy some useful structures or the functional layers and cause irreparable surface defects, particularly optical devices [164]. Apart from these conventional welding methods, flash light welding technology has been developed to address the high temperature intolerance of flexible substrates. At the same time organic solvents such as hydroxypropyl methylcellulose (HPMC) binders can be removed while effectively soldering silver wires (Fig. 8d) [156]. Moreover, Liu et al. [159] proposed another interesting approach to weld junctions via a self-limited cold-nanowelding technique in virtue of powerful capillary force at the nanoscale, hence also called capillary-force-induced welding (Fig. 8e). On nanoscale, the pressure between two contacting particles induced by capillary force can achieve MPa to GPa level, which is comparable to the pressure of mechanical pressing for the welding of AgNWs. This welding can result in significantly reduced network resistance and improved mechanical flexibility, without inducing any significant change in the optical transmittance for transparent application.

Chemical welding techniques is another typical welding methods, which reduce the contact resistance by redepositing Ag^+^ ions near the junctions via the redox reaction with the help of chemical reagent (e.g., H_2_O_2_ [165], N_2_H_4_ [166], sodium halide salts [161], ionic liquid [167]). Unlike other types of welding, chemical welding method does not require any external energy because it takes place in a solution environment. Cho and co-workers developed a method of chemically welding AgNWs using an aqueous solution containing sodium halide salts (NaF, NaCl, NaBr, or NaI) [161]. The halide welding dramatically reduced the sheet resistance of the AgNWs because of the strong fusion among nanowires at each junction and enhanced the mechanical flexibility of AgNWs. The optimized AgNWs electrodes exhibited a sheet resistance of 9.3 Ω sq^−1^ at an optical transmittance of 92%. As opposed to thermal and plasmonic welding techniques, the chemical welding could be applied to AgNWs films with a variety of deposition densities because the halide ions uniformly contacted the surface or junction regions. Recently, Li’s group employed an ionic liquid (IL)-type reducing agent containing Cl^−^ and a dihydroxyl group to control the reduction process of silver during welding process in wire–wire junctions precisely [167]. This delicate welding technique can facilitate an atomic-level contact between the AgNWs and the reduced Ag, which can decrease the sheet resistance, and enhanced the mechanical stability of AgNWs in like manner.

Another soldering technique differ from welding techniques in terms of the use of additives (a conductive solder) to fuse the junctions. Apart from the reactive silver ink as additives, PEDOT: PSS [168], GO [169], MXene [160] are employed as additives for soldering. Simultaneously, combining the above-mentioned ways for reducing junction resistance not only reduces resistance further, but also makes it better suited for specific extreme processing conditions. A representative example is provided by Chen et al., who fabricate a transparent and conductive AgNWs film with both high EMI shielding performance and high light transmittance by a soldering with MXene and cold-nanowelding technique (Fig. 8e) [160]. This capillary-force-induced welding method can enhance mechanical strength to the soldered junctions as well as significantly reduced contact resistance during the drying process, without the requirement of any treatment with heat or force (Fig. 8f, g). Likewise, mechanical roll process with high temperature is prevalent to sinter the AgNWs, which combine the mechanical welding and thermal welding [158, 170].

When considering the drawbacks of AgNWs used for shielding materials, the most notable ones are high cost, susceptibility to oxidation, and poor stability. The preparation of AgNWs involves a variety of chemical reagents and equipment, with multiple preparation parameters requiring strict control, all of which contribute to their elevated cost [148]. Furthermore, the chemical properties of the AgNW surface are highly susceptible to environmental oxidation, leading to decreased electrical conductivity upon exposure to air. To mitigate this issue, measures such as utilizing chemical modifiers or polymer coating agents can be implemented to safeguard the surface of AgNWs [171]. Additionally, the stability of AgNWs is suboptimal, making them easily influenced by environmental factors. To address this concern, approaches such as utilizing stabilizers to manage surface chemical reactions or adjusting their morphology, structure, and surface chemistry can be pursued to improve their stability.

##### CuNWs

CuNWs with outstanding electrical conductivity (~ 5.7 × 10^7^ S m^−1^) and ease of manufacture are also applied as conductive metal nanofillers for EMI shielding composites [172, 173]. Since CuNWs have remarkable electrical conductivity as well, the conductive network they produce has a similar EM shielding process to that of silver nanowires [174]. Likewise, the elastic EM shielding materials created employing CuNWs as conductive fillers can also have good tensile properties [175], strong EMI SE stability, and effective compression recovery [176]. And the wire-to-wire lap joint also produces a junction resistance that is significantly larger than the intrinsic resistance when CuNWs form a percolation network, therefore the junction welding procedure is equally crucial [177, 178]. Significantly, they are more affordable than AgNWs. However, bare CuNWs have a harmful propensity for oxidizing when exposed to air, which would cause a rapid decline in performance [27].

### 2D Material

#### Graphene

Graphene is the first two-dimensional (2D) atomic crystal available to us, which possesses an impressive range of material properties, including excellent electrical and thermal conductivity, mechanical stiffness, strength, and elasticity [179, 180]. The common methods to produce graphene powders are mechanical exfoliation, redox and SiC epitaxial growth, while the method to produce thin films is usually chemical vapor deposition (CVD). Due to the advantages displayed by their crystal flaws, reduced graphene oxide (rGO) is used in the majority of EM function studies [28]. Notably, a *sp*^3^-hybridized region for enhanced dielectric polarization, grafted function groups for simple compositing and structuring, and established and large-scale production technology are some of these benefits [181]. Graphene oxide (GO), which also uses graphene as a basic material, is hardly utilized in EMI shielding. In part that is because these resultant composites often display a low electrical conductivity and an insufficient EMI SE level, which is mainly because of the high structural defects (oxygen functional groups, heteroatoms, dangling bonds and vacancies etc.) of GOs caused by the oxidative process [182]. Even though GO is not used as the ultimate EM protection material in most cases, GO is usually employed as popular building precursors via reduction because of abundant oxygen groups associated with excellent dispersibility in aqueous solutions. But while the electrical conductivity of GO-based composites can be partially enhanced through chemical or thermal reduction processes to yield rGO, there is still a gap toward practical applications [183]. To boost the EMI shielding performance of graphene composites under the premise of ensuring the rational deformability of those, there have been two main ways as follows:

(i) Grafting other materials

There are essentially two types of additional modified materials that were incorporated into the graphene system: one is the high-conductivity materials such as CNTs [182, 184, 185], carbon nanohorn (CNH) [186], AgNPs [74, 187], AgNWs [142, 188], MXene [184, 188], that can substantially mention the conductive loss of graphene EMI shielding materials; the other is the nanomagnets such as Fe_3_O_4_ [31, 186], FeCo [189] nanoparticles that can magnetize graphene materials, hence boosting the magnetic loss. Clear evidence proves that the introduction of pristine SWCNT creates an exceptional 3D conducting and reinforcement skeleton, which can not only provide double fast channels for electron transport but also effectively transfer external load [183]. Accordingly, the resultant composite first achieves the EMI SE of a rather high 31 dB over the X-band frequency range and an intriguing conductivity of 120 S m^−1^ with an ultralow loading of 0.28 wt%. Guided by the foregoing, graphene composites were decorated with other foregoing high-conductivity nanomaterials to prepare various graphene-based hybrid EMI shielding elastic materials with multiple-percolation networks. For example, Ti_3_C_2_T_*x*_/rGO hybrid aerogel presented a higher EMI SE of over 54.8 dB than pure rGO aerogel, which possessed good compression resilience (~ 30% strain, 100^th^) as well [190]. Likewise, AgNWs/rGO and AgNPs/rGO hybrid composites have exceptional EMI SE over the X-Band regime of ~ 45.2 and ~ 67.3 dB, respectively; these values are better than those of rGO-only shielding armors [187, 191]. In addition to stabilizing the 3D graphene shape and absorbing some external pressures, the aforementioned nanosilver can also increase the overall deformability.ii) Heteroatom doping

One of the best and most direct ways to change the electrical conductivity and EMI SE of graphene is doping [192]. Doped graphene has exceptional properties as a result of its large specific surface area, high density of defects, strong electrical and thermal conductivity, and narrow tunable band gap (due to doping) [193]. Structural defects in the carbon lattice due to doping also help to decrease microwave energy by scattering and multiple internal reflections [192]. More importantly, this structural design does not cut the good mechanical stiffness, strength, and elasticity of graphene so drastically that it overly affects the overall deformation.

**Fig. 9:**
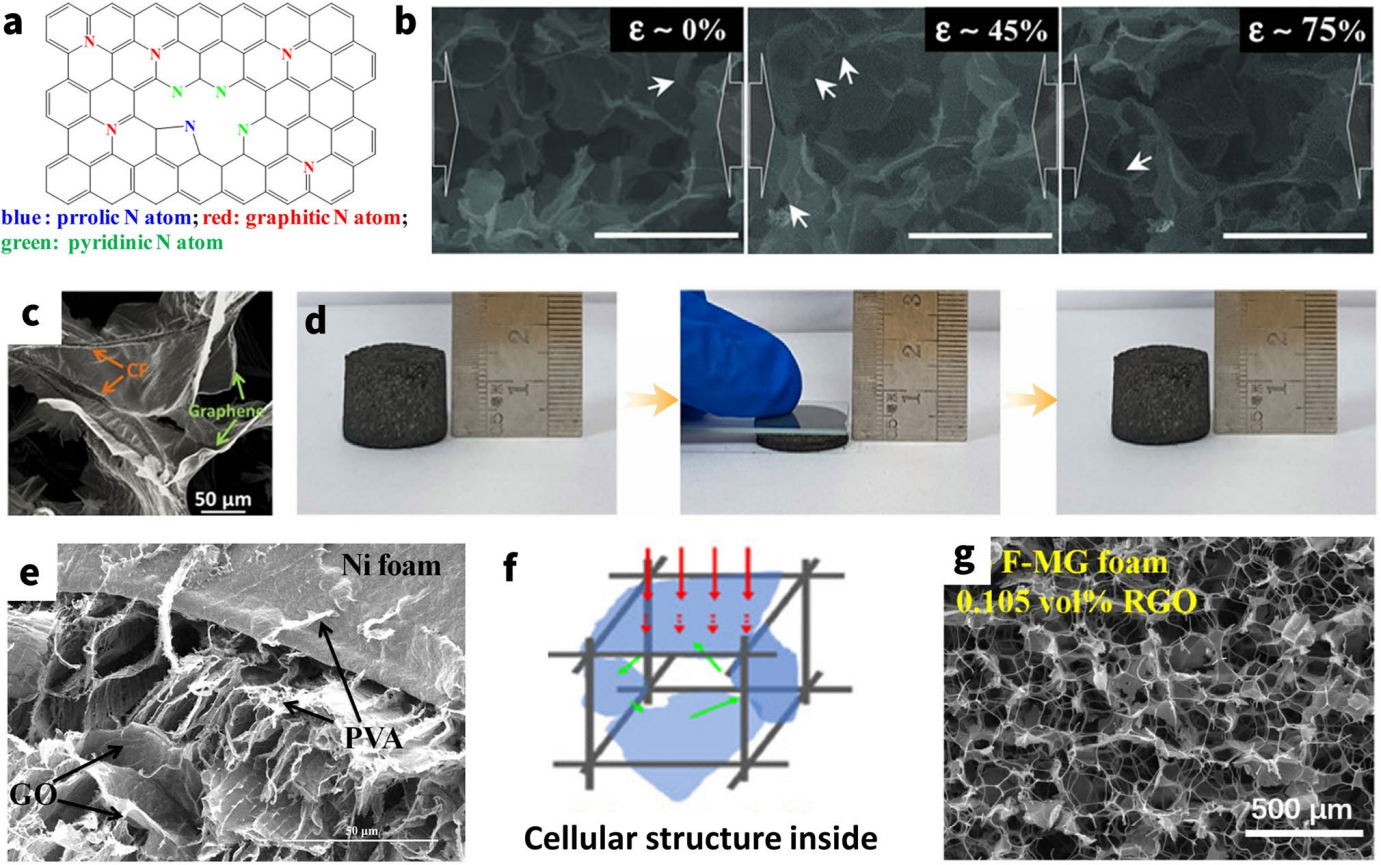
**a** Schematic map of N-doped graphene. **b** SEM images of loading states of rGO aerogel under uniaxial compression (scale bar = 250 mm). **c** SEM image of graphene/CNF aerogel. **d** Digital images of (Left) rGO/CNF-PMMA aerogel (Center) being loaded and (Right) released under a strain of 80%. **e** SEM image of GO/PVA/ Ni foam. **f** Schematic illustration of multi-reflection of EM waves in the cellular structure built by stretched rGO sheets in graphene foam. **g** SEM image of “obstacle walls” graphene foams fabricated by fluid-assisted method. **a** Reproduced with permission [199]. Copyright 2019, Springer Science Business Media, LLC, part of Springer Nature.** b** Reproduced with permission [212]. Copyright 2015, Wiley-VCH.** c** Reproduced with permission [217]. Copyright 2017, Elsevier Ltd.** d** Reproduced with permission [288]. Copyright 2021, Elsevier Ltd and Techna Group S.r.l.** e** Reproduced under the terms of the CC-BY Creative Commons Attribution 4.0 Generic license (https://creativecommons.org/licenses/by/4.0) [214].** f, g** Reproduced with permission [179]. Copyright 2019, The Authors

Typically, boron (B) [194], nitrogen (N) [195, 196], phosphorus (P) [197], and sulfur (S) [198] as doping element for pristine graphene have all been studied with the purpose of designing improved EMI shielding materials. For instance, Lin et al. [199] fabricated the 6.6-μm-thick nitrogen-doping rGO film, which possesses ultrahigh electrical conductivity of 8796 S cm^−1^, leading to outstanding EMI SE (~ 58.5 dB) and the SSE/t (43,902 dB cm^2^ g^−1^) (Fig. 9a). And the EMI SE of ~ 48 dB and conductivity of 1575 S cm^−1^ of pristine rGO film are both much less than those of nitrogen-doping rGO film. Interestingly, some special nitrogen-doping sources served as a nitrogen dopant and reducing agent in the hydrothermal reaction process, but also played a role as modifier in the self-assemble formation process of the porous hydrogels [200, 201]. Moon et al. [193] employed hexamethylenetetramine (HMTA) as a reducer and a nitrogen source to prepare ultralight *N*-doped rGO aerogels with a density of ~ 3.20 mg cm^−3^. During hydrolysis in an aqueous solution, however, HMTA releases ammonia and hydroxide ion [202]. An abundance of hydroxide ion and ammonia can reduce graphene oxide to rGO by removing oxygen-containing functional groups and can simultaneously introduce nitrogen atoms into the graphene skeleton by substituting carbon atoms, respectively [203, 204]. Besides, as illustrated in Fig. 9b, the nitrogen-doping rGO aerogels have a stable structure and good compression resilience. And the conductivity of the nitrogen-doping rGO aerogel upon unloading was ~ 11.74 S m^−1^, whereas under ~ 80% compressive strain (*ε*), it was ~ 704.23 S m^−1^. This remarkable aerogel has great potential in the field of elastic EMI shielding due to the huge advantages described above.

There has also been a great deal of effort to obtain highly deformable graphene materials for EMI shielding. The resulting 3D skeletons (such as aerogel, hydrogel, foam, and sponge) are more ideal for use in compression scenarios because of the natural sheet-like and in-plane structures of graphene, such as piezoresistive sensor [205] compressible EMI shielding armors [206, 207], rather than stretchable application. Additionally, for each of these application scenarios, graphene must be resilient, repeatable, and able to endure numerous cycles of compression and release. Moreover, the mechanical properties of graphene aerogels are mainly controlled by the strong and robust sheet-to-sheet interfaces, facilitating the efficient load transfer, impacted by inherent van der Waals forces between layers. However, the resultant graphene-only 3D aerogel skeletons, formed by self-assembly methods, still have generally failed to exhibit high compressive strain (more than 80%) and excellent fatigue resistance owing to the instability of the three-dimensional structure and fragile nature of graphene [208]. Significantly, the mechanical properties may also be influenced by hydrogen bonding, or even covalent bonding provided by insulating polymers or amorphous carbon with low conductivity, which depended on other materials for synthesis support of aerogels [209]. Therefore, bridging the polymeric materials during “sol–gel” process, such as polyimide (PI) [124, 209], polymethyl methacrylate (PMMA) [208, 210], CNF [208, 211], aramid nanofiber (ANF) [190], with the rGO sheets can effectively enhance the mechanical properties and meet the demand for repeated rebound by creating new bonds. Wong’s group fabricated rGO/cellulose fiber (CF) hybrid aerogel through lyophilization and carbonization process [212]. The resultant aerogel exhibits high EMI SE of ~ 47.8 dB after annealing at 1000 °C. Moreover, the wrinkled topology of CF (see orange arrow in Fig. 9c) caused by thermal treatment plays an important role in promoting the mechanical interlocking and load transfer with graphene sheets, which could enhance the mechanical properties of rGO/CF sponge. Moreover, the hybrid aerogel possesses excellent mechanical resilience even with large strain (80% reversible compressibility) and outstanding cycling stability. Guided by the foregoing, Liao et al. [208] added PMMA to the rGO/CNF aerogel to enhance the mechanical elasticity. The resultant rGO-CNF/PMMA exhibits super compressibility and excellent elasticity, and can resist an extreme compressive strain of 99.3% while maintaining 92.6% of the height retention after 5000 cycles at the strain of 80% (Fig. 9d). The elasticity and fatigue resistance can also be significantly enhanced by the carbonized version of the aforementioned polymer when it is used as an enhancer. Although the “sol–gel” method for 3D rGO skeletons is simple, serious shrinkage and deformation accompany the reduction of GO, resulting in the obtained 3D rGO skeletons are inhomogeneous and very fragile, which is very detrimental to the backfill of polymer matrix and the shaping processing of polymer composites [142].

Additionally, there are some examples of how to create porous compressible EMI shielding armors utilizing different kinds of commercial sponges as templates, including PU and melamine. Polymer-based sponges, which have good strength and porous structure, are usually used as cleaning, soundproofing, and packaging materials. With the introduction of sponge, the 3D network characteristic of sponge and the advantages of graphene sheets are combined. Recently, graphene composites based on PU sponge have been developed by simple solution dip-coating by Zhen’s group [213]. The resultant graphene/PU foams had a density as low as ∼0.027–0.030 g cm^−3^ and possessed good comprehensive EMI shielding performance of ~ 57.7 dB in the X-band range together with an absorption-dominant mechanism, possibly due to both conductive dissipation and multiple reflections and scattering of EM waves by the inside 3D conductive graphene network. Moreover, the average SE total of the graphene/PU foams with thicknesses of ∼6 cm did not show an observable decrease during 50 cycles with different compressive strains (undergoing an extreme strain of 75%), indicating an excellent cycling stability. Inspired by this strategy, Fu et al. [32] successfully fabricated graphene nanosheets (GNSs) wrapped MF (GNSs@MF) by repeated dip-drying method using MF skeleton as substrate. Importantly, the excellent EMI SE of 35.6 dB in the X-band is reliable even the prepared composites undergoing vigorous physical damages and long-term compression cycles due to the protection of TPU layer and inherent elasticity of MF. However, due to the unsatisfactory adhesion of graphene to the polymer foam matrix, the coating rGO nanosheets may severely fall off during the compression, and thus the EMI shielding performance may be degraded [214, 215].

Ni foam have also attracted considerable interest as skeleton materials. Compared with polymer-based sponges, metal Ni foam possesses considerably high conductivity (*σ*_bulk Ni_≈1.443 × 10^7^ S m^−1^) and permeability, reflecting the large number of incoming EM waves, which frequently serves as rational EMI shielding armor [216]. Combined with rGO, the synergistic effect of dielectric and magnetic loss improves the EMI SE of the materials. Recently, Li et al. [217] have successfully prepared Ni foam/GO/PVA composite aerogels were by a freeze-drying method (Fig. 9e). The maximum EMI SE of resultant composite can reach 87 dB at the thickness of 2.0 mm due to synergistic effect of Ni foam and GO. Simultaneously, the deformation is mainly elastic deformation when the compression strain below 5%, which can be recovered after unloading. Unlike polymer foam templates, Ni foam can also act as a framework to prepare graphene foam by CVD method due to its high temperature resistance. Wang et al. [218] fabricated a graphene foam by CVD and the EMI SE of resultant composites was 32 dB with 0.4 wt% graphene. Under the stress of 1000 kPa, the EMI shielding coefficient of the proposed composite was 25 dB, which was reduced by 21.9%. In some processes, the Ni foam is used as an intermediate template to form the pure graphene foam, rather than accompanying the graphene to form the final EMI shielding material. After the graphene foam is prepared, it is eliminated by etching with solutions such as FeCl_3_ and HCl [187, 218].

Another highlight in the research and development in this field is the nanostructure manipulation of graphene sheets. In most works, graphene nanosheets were indeed wrapped rather than stretched (as showed in Fig. 9f), on the skeletons of foams to hinder the propagation of EM waves [219, 220]. The coated rGO nanosheets may, however, significantly come off under compression due to the inadequate adherence of graphene to the polymer foam matrix. In particular, for the polymer-based foam templates, the EW leakage would easily occur once most of the skeletons are not wrapped, leading to a weak EMI shielding performance [221]. For obtaining stretched-graphene nanosheets, Guo et al. [214] employed a fluid-assisted method to make graphene nanosheets cover on the pore of sponge formed the “obstacle walls” (Fig. 9f). The resultant foam demonstrates the EMI SE of 37.2 dB with rGO content of 0.105 vol%, and the specific EMI SE up to 3410 dB·cm^3^ g^−1^ with the density as low as 0.011 g cm^−3^. Notably, stretched-graphene foam presented a higher EMI SE of over 37 dB than that of wrapped-graphene composite foam (~ 7 dB) (Fig. 9g). The stretched composite foam also exhibited robust mechanical property, and a small amount EMI SE reduction was observed with 50 cyclic of compression.

**Fig. 10:**
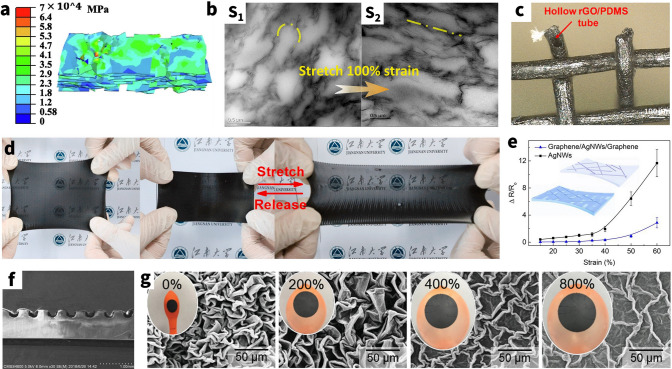
Graphene-based stretchable EMI shielding composites. **a** FEA of laminated graphene film under 6% tensile strain. Reproduced with permission [227]. Copyright 2021, Elsevier B.V.** b** TEM images of the GNR-6 composites before (S1) and after (S2) the rubber permanent deformation with 100% strain (15 mm stretched length). Reproduced under the terms of the CC-BY Creative Commons Attribution 4.0 Generic license (https://creativecommons.org/licenses/by/4.0) [223].** c** Optical image of rGWF/PDMS composites. Reproduced with permission [224]. Copyright 2019, IOP Publishing Ltd.** d **Digital photographs of graphene/PDMS lattice film. Reproduced with permission [225]. Copyright 2021, Elsevier Ltd.** e** Resistance as a function of strain for the graphene/AgNWs/graphene film and the AgNW-only network. Insets image: the schematic diagrams showing the stretched samples. Reproduced with permission [226]. Copyright 2021, This is a US government work and not under copyright protection in the USA; foreign copyright protection may apply.** f** Cross-sectional SEM images of graphene/PDMS composite. Reproduced with permission [199]. Copyright 2019, Springer Science Business Media, LLC, part of Springer Nature.** g** SEM images and digital photos of crumple-textured GO/MXene/SWCNT coating under various areal strains. Reproduced with permission [184]. Copyright 2019, Wiley-VCH

When used as a stretchable EMI shielding material, the brittle percolation network structure of graphene, which is attributable to the low aspect ratio, stiff characteristics, and in-plane structures, tends to shatter during deformation and hence loses its EMI shielding capabilities (Fig. 10a). The compressible graphene EMI shielding material discussed above has a porous structure that makes it unsuitable for significant bending and stretching since this would cause the already brittle conducting network to disintegrate [188, 222]. Not only that, but the graphene-coated film is also unable to withstand the damage caused by a large pull-up. Currently, there has been an influx of exploration on how to enhance the tensile properties of graphene composites. The relevant methods are summarized as follows:

i) Enhancing the conductive path

Generally, graphene is homogeneously dispersed in the matrix, and the constituted conductive path is more fragile due to the relatively low concentration of graphene per unit area. Therefore, localizing graphene, in other words, increasing the concentration used for constructing the conductive pathways, can mechanically enhance the conductivity stability of graphene EMI shielding films after stretching strain, thus fundamentally solving the problem of decreased EMI SE generated by stretching. For example, Wang et al. [223] prepared the rGO/Fe_3_O_4_/natural rubber composites with a segregated network was by electrostatic self-assembly. As a result, the conductive particles formed by rGO-Fe_3_O_4_ are bound around the small pores formed by natural rubber to act as pore walls, increasing the concentration of conductive filler within the pore walls (Fig. 10b). In comparison with the identical structure manufactured from rGO/natural rubber (GNR) composites, the EMI shielding property of the resulting composites is more stable during tensile deformation and long-term cycling conditions and has a higher sensitivity to stretch strain. The EMI SE value of GNR composites reduces by no more than 2.9% under different tensile permanent deformation, cyclic stretching, and cyclic bending conditions, while that of GNR composites reduces by approximately 16% in the worst case. Chen et al. [224] fabricated rGO woven fabrics (rGWF)/ PDMS composites through a facile template-directed reduction method followed by dip coating. The fabricated composite possesses a highly ordered and hierarchical porous structure, containing the unique hollow tubes constructed by 3D interconnected dense graphene networks (Fig. 10c). The unique porous structure containing high-quality graphene architecture makes the composite exceptional EMI shielding properties. The composite containing four layers of rGWF delivers a remarkable EMI SE of 46 dB and a specific SE of 295 dB cm^3^ g^−1^. Apart from this property, the composite also exhibits excellent durability and is capable of retaining over 94% of the original SE after 100 stretching-releasing cycles. Wang et al. [225] fabricated rGO/PDMS lattices through the 3D printing technique (Fig. 10d). Benefiting from the unique 3D interconnected and robust conductive network, the resultant lattice delivers excellent stretchability of 130%, tunable EMI SE as high as 45 dB, along with exceptional durability, showing over 90% retention of EMI SE even after 200 cycles of repeated stretching and releasing at strains up to 100%. In addition, the lattice exhibits outstanding shielding stability, because the deformation of lattice structure effectively shares the external strain, and the filaments perpendicular to the loading direction act as stabilizing layers preventing the steep resistance changes. As described above, these exceptional combinations of mechanical properties and EMI shielding performance of the composite provide a brand-new perspective for ultra-stretchable graphene EMI armor.

ii) Incorporating other nanoconductors

A nanofiller can bridge adjacent graphene nanosheets to provide additional conductive paths, which minimizes the effect of conducting path breaks when graphene receives external deformation. Among them, the one-dimensional materials such as AgNWs, CuNWs, and CNTs mentioned in the previous section can bring unexpected effects to the construction of ultra-stretchable graphene EMI shielding film (Fig. 10e) [185, 226].

iii) Optimizing the graphene geometric morphology

Graphene nanoribbon (GNR) immediately attracted worldwide attention since appearance as a bridge between graphene and CNT [227]. Theoretically, due to the large aspect ratio, GNR are considered to be the most suitable graphene-based electronic fillers for stretchable composites as they can easily form the percolation network structures within matrix [228]. Park and coworkers synthesized GNR by unzipping MWCNTs and the resultant PU composites [229]. The EMI SE of GNR/TPU composite was 24.9 dB, which is considerably greater than that of MWCNT/TPU composite (9.3 dB) at 8.2 vol%. Moreover, the stretched composite foam also exhibited robust mechanical property, whose stretch strain over 100%.

In addition to the above three methods to enhance the intrinsic stretchability of graphene composites, pretreatment of the substrate can also achieve the overall structural stretchability enhancement, which can extend the stretchable deformation range and enhance the stretch-invariant electrical conductivities stability [135]. Generally, pre-stretching the stretchable polymer substrate to form a wavy wrinkle structure can effectively improve the material tensile properties at a later stage, which is also the more popular pretreatment method (Fig. 10f). For example, Lin et al. [199] obtained the rGO/PDMS stretchable shielding composite by fixing the rGO film on the pre-stretched wavy substrate, which exhibited the constant EMI SE of 56.3 dB after repeated stretching. The pre-stretched wavy substrate allows the multilayer graphene film to achieve wavy structure after strain release, which is capable of bearing tensile strain up to 32.6%. Furthermore, this special pleated surface also facilitates the attenuation effect of microwaves, and the increase in pleats at high pre-stretching is more prominent in the attenuation effect of microwaves [230].

Hybrid methods combining the four improvement methods mentioned above has also been reported for enhancing the stretchability of graphene EMI armor. For example, Li et al. [184] fabricated an ultra-stretchable EMI shielding composite with a hierarchical conductive system by depositing a crumple-textured coating composed of GO, 2D Ti_3_C_2_T_*x*_ nanosheets and SWCNTs onto latex, which can be fashioned into high-performance EMI shields. The resulting GO-MXene-SWCNT (S-MXene)/latex devices have the capacity to sustain up to an 800% areal strain and exhibit strain-insensitive resistance profiles during a 500-cycle fatigue test (Fig. 10g).

#### MXene

**Fig. 11:**
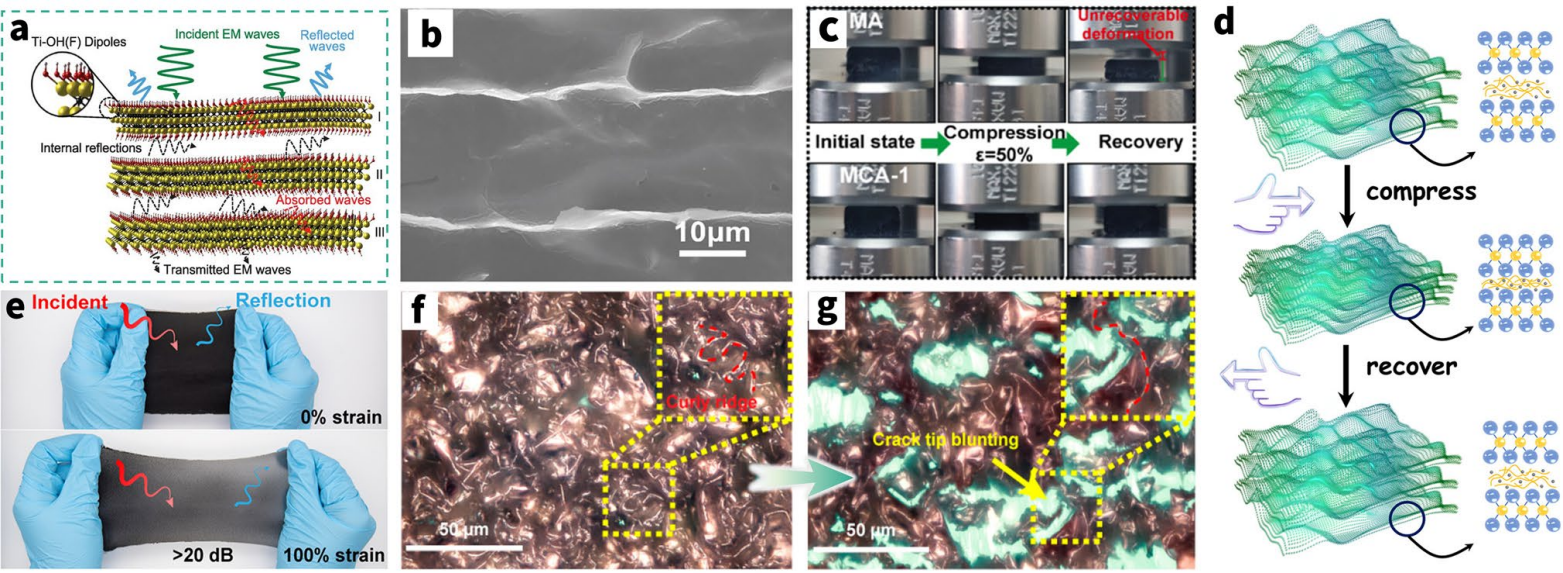
**a** Proposed EMI shielding mechanism of MXene flakes. Reproduced with permission [237]. Copyright 2016, American Association for the Advancement of Science.** b** High-magnification SEM image of MXene/SA-PDMS composite channels. Reproduced with permission [289]. Copyright 2019, Elsevier B.V.** c** Experimental snapshots of the first compression cycle of MXene aerogel (MA) and MXene-CNTs aerogel (MCA-1), respectively. Reproduced with permission [290]. Copyright 2021, American Chemical Society.** d** Schematic inner-microstructure changes of the MXene composites during compress—recover cycles. Reproduced with permission [232]. Copyright 2022, American Chemical Society.** e** Digital photographs of the MXene/PDMS film for EMI shielding under stretching deformations. The surface morphology of pre-stretching-formed ridge structures of MXene/PDMS:** f** initial state and** g** final state with a tensile strain of 100%.** e**–**g** Reproduced with permission [243]. Copyright 2021, American Chemical Society

MXenes are a unique family of two-dimensional (2D) transition metal carbides and/or nitrides with the formula M_*n*+1_X_*n*_T_*x*_, where M is an early transition metal (e.g., Ti, Zr, V, Nb, Ta, or Mo) and *X* is carbon and/or nitrogen. Owing to the aqueous medium used during synthesis, MXene flakes are terminated with surface moieties (T_*x*_), such as a mixture of –OH, =O, and –F [231, 232]. MXenes may intercalate organic molecules and ions, which makes them a viable choice for usage in polymer composites for EMI shielding due to their metallic conductivity, high mechanical characteristics, and hydrophilicity. About 20 different MXenes have already been reported [233, 234]. Among various MXenes, Ti_3_C_2_T_*x*_ has demonstrated considerable potential for EM functions due to its ultrahigh conductivity and abundant surface functional groups and defects (Fig. 11a) [235]. Ti_3_C_2_T_*x*_ films have shown the highest conductivity among all the MXenes studied so far, and it was assumed that they offer the best shielding properties. Yury’s group first synthesis Ti_3_C_2_T_*x*_ film with an EMI SE value of 92.0 dB at an extremely small thickness of 45 µm; these numbers are superior to those that have been reported for graphite, graphene, CNTs, and metals. From that point on, this marvelous research ignites academia’s enthusiasm for MXene-based superefficient shielding armors with surface treatment, micro-/macrostructure engineering, corresponding composites design and various matrix options.

From the point of view of the morphological properties of the material itself and the mechanical strength, the specific usage and areas in need of improvement of MXene and graphene, which also has a two-dimensional structure, are very similar in tensile/compression fields [235]. In contrast, MXene materials are more often used in the preparation of high-performance compressed EMI shielding materials due to their accordion-like multilayer structure. Self-assembly and the template approach are still the most popular ways to construct compressed MXene during the preparation phase [236]. The complexity of the two processes and the degree to which auxiliary materials are required for sample production varies, which is eventually reflected in the density and durability of the compressible MXene composites. Statistically, freeze-drying is the most pervasively used means of constructing 3D MXene, attributed to the ability to effectively avoid MXene agglomeration and to spontaneously form lightweight porous compressible materials with easy EM waves absorption [237, 238]. For example, Wu et al. [239] fabricated the lightweight MXene/sodium alginate (SA)-PDMS hybrid aerogel via freeze-drying with an outstanding conductivity of 2211 S m^−1^ and a high average EMI SE of 70.5 dB (Fig. 11b). Furthermore, the PDMS-coated MXene foam with 6.1 wt% of MXene reserves its high EMI SE of 48.2 dB after 500 compression-release cycles, which demonstrated it possessed excellent compressibility and durability. Likewise, the construction of elastic MXene aerogels usually requires the assistance of polymers or low-dimensional nanomaterials that could interact with MXene by van der Waals forces, hydrogen bonding, or covalent bonding (Fig. 11c, d) [237]. Otherwise, MXene-only aerogel cannot recover to initial state (~ 100% height) and thus induce to do harm to its inner nanostructure.

Notably, the self-supporting films formed of MXene alone are more brittle, extremely fracture-prone, and incapable of performing the duty of large-scale stretching for stretchable MXene composites. Therefore, methods to enhance the tensile properties similar to stretchable graphene composites are needed, including but not limited to grafting other stretchable nanoconductor [240, 241], introducing stretchable-shaped structures [242], etc. Chen et al. [243] prepared the Ti_3_C_2_T_*x*_ MXene/PDMS films by constructing wrinkled MXene patterns on a flexible PDMS substrate to create a hierarchical surface with primary and secondary surface wrinkles (Fig. 11e-g). The self-controlled microcracks created in the valley domains of the hierarchical film via a nonuniform deformation during pre-stretching/releasing cycles endow the hierarchical MXene/PDMS film with a high stretchability (100%), strain-invariant EMI SE (~ 30 dB at a tensile strain of 50%), and stable SE over a 1000-cycle fatigue measurement. Zhang et al. [240] fabricated EMI shielding textile based on hybrid Ti_3_C_2_T_*x*_ MXene and other 1D CNTs coated TPU non-woven fabric via dip-coating approach and pre-stretching method. The synergistic effects of carbon nanotubes and stretchable structures in MXene/CNTs conductive layer are beneficial for denser microcrack structure and more significant bridging effect, causing stable EMI shielding under stretching (~ 25 dB under 50% strain over 1000 cycles).

Interestingly, unlike the two-dimensional monolayer structure of graphene, MXene can be classified as either multilayer structure (ML-MXene) or few-layer or single-layer structure (FL-MXene) depending on the exfoliation method. Due to the fluctuation in the structure’s anisotropy and the distinction between the values of in-plane and out-of-plane conductivity, this must be discussed individually [244, 245]. The main differences in EMI shielding can be broadly divided into the following two items.

First off, FL-MXene has a greater specific surface area, which makes it easier for a complete conductive network to form. The stability of the conductive network in the stretched condition may be maintained more easily due to the wide FL-MXene layer-to-layer contact area [15]. However, the large specific surface area can cause FL-MXene to be extremely prone to stacking and agglomeration. In contrast, the dispersion of ML-MXene solution is significantly better.

Secondly, in the highly filled state, the imaginary part of the dielectric constant *ε*″ of FL-MXene is much higher than that of ML-MXene due to its better conductive properties. Further, it is also shown that FL-MXene possesses stronger EM loss capability. Recently, Ma’s group demonstrated that 3D Ti_3_C_2_T_*x*_ MXene aerogel with the ~ 40 wt% filler amount of FL-Ti_3_C_2_T_*x*_ has an average *ε*″value of ~ 200, while the *ε*″ value of ML-Ti_3_C_2_T_*x*_ is merely ~ 1 with same filler amount [246].

Besides, versatile chemical transformation of surface functional groups in MXenes is one of the most significant ways to improve the performance of MXene [247]. These modified MXenes show distinctive structural and electronic properties, whose surface groups also control superconductivity of extraordinary MXenes. However, there has been less research in this direction in the field of EMI shielding, probably because the modification process is complex and the conductivity of MXene itself is sufficient for shielding. Even so, the modification of the layer spacing and conductivity should provide better protection against EMI interference, which is one of the promising works [248].

Due to severe agglomeration and poor filler-matrix bonding, the drawback of nanofillers is that high loading can drastically reduce the mechanical flexibility and processability of composites. These nanofillers are both expensive and challenging to manufacture on a big scale. For these nanomaterials, a laborious purification or functionalization process is typically required.

### Other Material

#### Liquid Metal

Liquid metals (LM) possess the ultrahigh electrical conductivity of the metal itself (~ 1.1 × 10^4^ S m^−1^ with volume ratio of 50%) and the superb deformability, especially stretchability (theoretically infinite), given by the liquid properties [23, 249, 250]. Early studies utilize Hg, which is toxic [251, 252]. Low toxicity liquid metals based on Ga metal have been studied, including Ga alloyed with In (EGaIn), and with In and Sn (Galinstan) [253]. Contrary to the delicate but fragile conductive paths created by conductive nanofillers, the conductive paths created in the EMI shielding materials by liquid metals are significantly stronger and do not easily break and fail with deformation. This makes them superior to the conductive nanofillers mentioned in the previous subsections [254–256]. Additionally, they can maintain metallic conductivity while being infinitely malleable, which has recently drawn a lot of interest to create composites with superior stability and conductivity to their solid filler-based competitors.

**Fig. 12:**
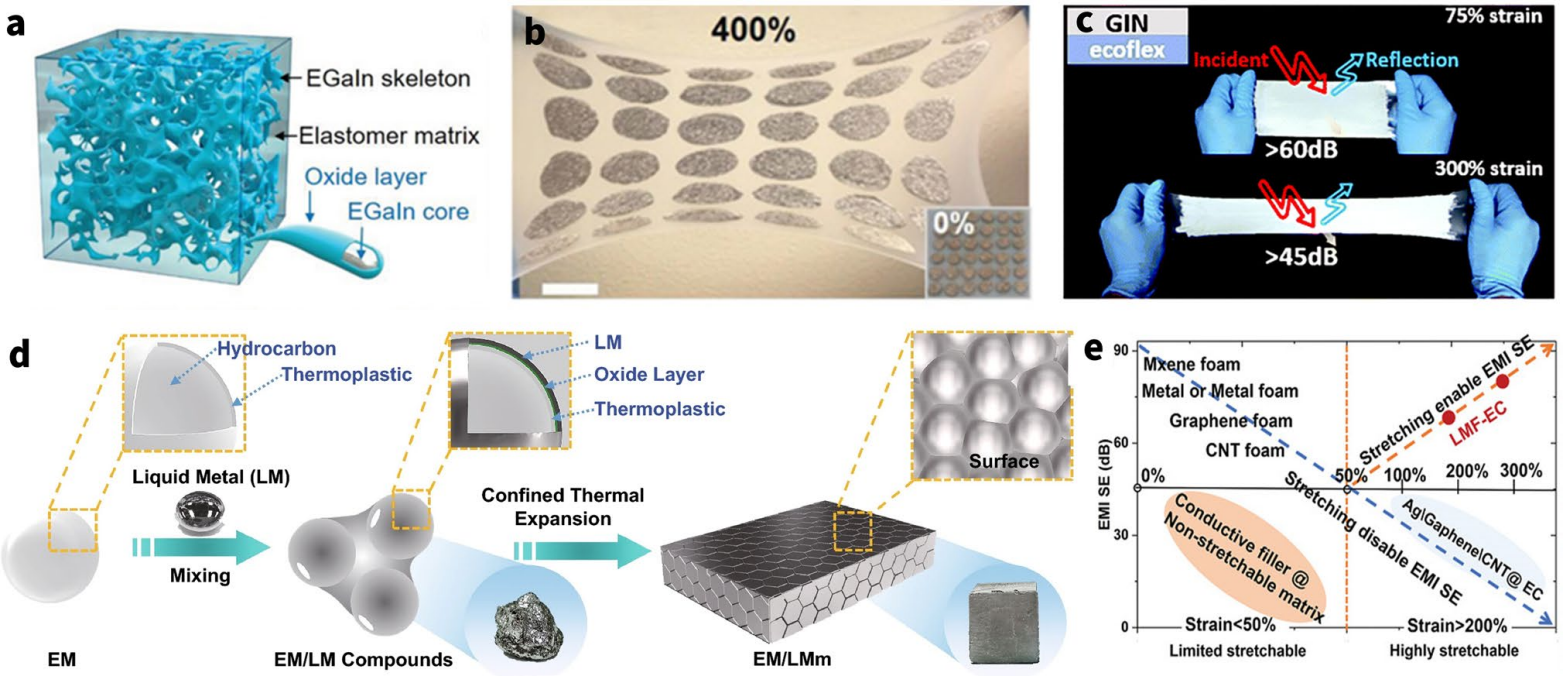
Liquid metal-based elastic EMI shielding composites. **a** Schematic of the 3D LM composite. **b** Photographs of a highly stretchable 3D LM composite film with patterns. Scale bar, 2 mm. **c** Digital photograph of GaIn-Ni particles painted on Ecoflex and a simple description of the shielding performance during stretching. **d** Schematic of expandable microsphere/LMm preparation and macroscopic features demonstration. **e** The comparison between LM-based elastic materials with the other EMI shielding materials. **a, b** Reproduced with permission [23]. Copyright 2017, Wiley-VCH.** c** Reproduced with permission [258]. Copyright 2019, The Royal Society of Chemistry.** d** Reproduced under the terms of the CC-BY Creative Commons Attribution 4.0 Generic license (https://creativecommons.org/licenses/by/4.0) [249].** e** Reproduced under the terms of the CC-BY Creative Commons Attribution 4.0 Generic license (https://creativecommons.org/licenses/by/4.0) [291]

Despite their very high conductivity and stretchability, there exists a major challenge in patterning liquid metals due to their ability to flow [257]. The typical approach for the preparation of the LM composites is to encapsulate LM micro-/nanonetworks into the elastomer matrix. Yao et al. [23] first fabricated a stretchable LM/Ecoflex composite with a 3D conductive network via sugar template method, which exhibited an obvious increase of EMI SE when stretched (from ~ 45 dB of pristine state to ~ 80 dB of the stretch to 400%) (Fig. 12a, b). Interestingly, stretching LM causes it to deform together with the surrounding matrix, which enhances electrical conductivities and EMI SE. And the excellent stretchability of LM-based elastic conductors should be attributed to the high deformability of LM, which matches well with the mechanical behavior of elastomer matrices and ensures continuous straight LM conductive paths even under very large strains. Zhang et al. [258] used the amalgam composed of GaIn_24.5_, NiNPs and gallium oxide (Ga_2_O_3_) as the conductive functional layer and Ecoflex as substrate to fabricate the highly stretchable composite film by coating techniques. This film demonstrated excellent EMI SE of over 75 dB of pristine state and ~ 60 dB even at strains of up to 75% at frequencies of 100 kHz–18 GHz (Fig. 12c). 3D printing of liquid metals has also been utilized to rationally assemble LM into an elastomer lattice for restrict the flow of LM more handily. Wang et al. [17] prepared the 3D interconnected LM/PDMS lattice skeleton by 3D printing, yielding the resultant composites high electrical conductivity (1.98 × 10^6^ S m^−1^), stretchability (180%), and EMI SE (72 dB). Furthermore, LM is not limited to shield the EMI in the form of inner filler in the matrix, instead of coating on the elastic polymer, thus dramatically reducing the usage amount [259]. Jia et al. [260] developed a LM coated conductive textile, which exhibits an outstanding EMI SE of 72.6 dB at a thickness of merely 0.35 mm while maintaining EMI SE of 66.0 and 52.4 dB under strains of 30 and 50%, respectively. The corresponding EMI SEs hold 91.7 and 80.3% retention after 5,000 stretching –releasing cycles, respectively. The superior and durable EMI SE should be ascribed to the perfect connectivity and good deformability of conductive LM networks.

Alternatively, the LM-based materials will be endowed with the capability of compression when changing textile matrix into another compressible polymer. Wong’s group succeeded in changing textile matrix into expandable microsphere and proposed an expandable microsphere/LM composite via confined thermal expansion process (Fig. 12d). And the monolith presents outstanding performance of lightweight like metallic aerogel (0.104 g cm^−1^), recorded SE (98.7 dB) over 8.2–40 GHz, super elasticity (90% strain) [261]. Notably, generic porous foams fabricated by other foaming method are also employed in the preparation of compressible LM composites [262].

As illustrated in Fig. 12e, LM-based elastic EMI shielding materials have superb stretchability and excellent EM waves shielding performance, especially compared to other nanofiller-based materials. However, there are still some problems that hinder the continued development of the LM, one of the more serious ones being the further worsening of the leakage problem at high fill volumes. Meanwhile, due to the limitations of liquid metal itself, it is challenging to create anisotropy of the counterpart and graft with other nanomaterials.

#### Conductive Polymer

Conductive polymers hold great potential for enabling new EMI shielding applications due to the intrinsic electrical conductivity and biocompatibility as well as the favorable tissue-like mechanical properties and durability derived from the polymeric nature [263, 264]. Conductive polymer shielding materials, which are based on stable molecular-level materials, are frequently utilized to build electronic devices with smooth interface requirements [265].

Amongst the family of conducting polymers, PEDOT:PSS has been extensively studied conducting polymer due to its high electrical conductivity, low density, and good environmental stability [266]. Since it can be dispersed in water and some organic solvents, it can be processed using solutions processing procedures. However, due to the rigid conjugated backbone, PEDOT:PSS films have very limited stretchability (Maximum strain ~ 5%) [267]. The method to make PEDOT:PSS more stretchy avoids the aggregation of other nanofillers by combining it with elastic polymer. Ouyang’s group prepared a stretchable PEDOT:PSS film by blending PEDOT:PSS with highly stretchable WPU [268]. The two polymers have good miscibility at a wide range of blending ratios. At a 20 wt% PEDOT:PSS loading, the composite films show a conductivity of 77 S m^−1^ and an elongation at break of about 32.5%. More interestingly, they exhibit a high EMI SE of about 62 dB over the X-band range at 0.15 mm. Apart from that, a plasticizer can also be used to create PEDOT:PSS that is extremely stretchy [267].

Furthermore, hydrogels made of soft (10–100 kPa Young's modulus) conducting polymers have been developed. A hydrogel solely made of PEDOT:PSS showed a high conductivity of 4100 S cm^−1^ and a stretchability of 60% [269]. Improved mechanical properties and EMI shielding performance have been obtained by the incorporation of another conductive fillers, such as MXene [270], ionic liquid [14], Fe_3_O_4_ [95]. Another form of PETDOT:PSS is aerogel, which will obtained by taking out of water from the hydrogel thoroughly [271]. It is portable and compressible to have a porous structure.

Another class of conducting polymers, polypyrrole (PPy), has also been used as a stretchable shielding material in addition to PEDOT: PSS. As a coating, the formation of a coated EMI shielding stretchable fabric can further enhance the EMI performance (~ 40 dB) without compromising the tensile properties (~ 25%) [101]. Composites with PDMS showed a high EMI SE (~ 21 dB) and high stretchability (~ 100%) [272]

#### Biomass-Derived Carbon Foam

**Fig. 13:**
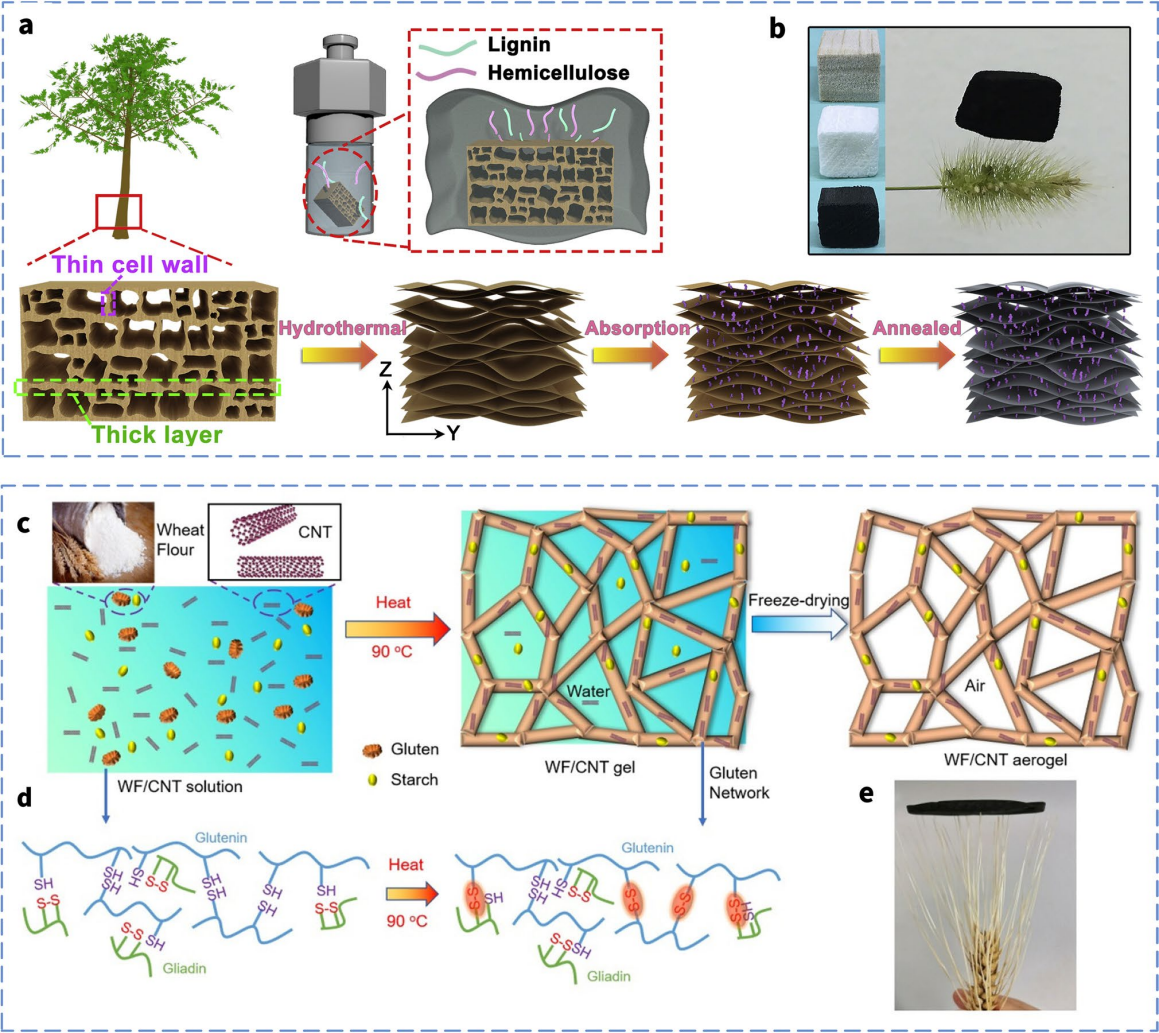
Biomass-derived carbon composites-based elastic EMI shielding composites. **a** Schematic illustration of the synthesis of the off/on switchable EMI shielding aerogel. **b** Digital photographs of natural wood (left, yellow), wood sponge (left, white), CB/wood sponge (left, black), and CB/wood aerogel (right, black). **c** Schematic diagram for preparation of wheat flour/CNT foam. **d** Schematic illustration for formation of the cross-linking backbone of gluten proteins. **e** Photograph of the different shapes of wheat flour/CNT foam. **a, b** Reproduced with permission [21]. Copyright 2021, Elsevier Inc.** c**–**e** Reproduced with permission [262]. Copyright 2021, American Chemical Society

As an alternative, carbon derived from biomass has been widely investigated with respect of EMI shielding due to their sustainable raw materials and unique conductive frameworks. Among the most important renewable resources are lignin, carbon, wood, wheat, and lather. Some biomass can use its own porous carbon skeleton, which not only avoids the foaming process but also has a stable porous structure that is less prone to collapse and has strong compression capabilities, to create the support structure of the new material [273, 274]. For example, when the supporting lignin is removed, the softened cellulose-based wood has a unique hierarchical porous structure and is also compressible (> 50%) (Fig. 13a, b). After carbonization, loading on different conductive fillers can achieve efficient EM protection, such as carbon black (25.5 dB) [21], CNT/MXene (~ 30 dB) [275], etc. Some other biomass can be interchanged with the nanofillers described above and, after carbonization, act as conductive pathways [276]. Chen et al. report herein a novel utilization of wheat flour with the introduction of CNTs to form an environmentally friendly wheat flour/CNT composite foam (Fig. 13c-e). This foam displayed a high elasticity (nearly 100% shape recovery), recyclable (5000 cycles), high EMI SE (~ 40 dB). Unfortunately, after carbonization, these biomass skeletons generally have low conductivity and poor EMI SE, requiring grafting of other high conductivity filler modifications.

## Summary and Future Perspectives

**Fig. 14:**
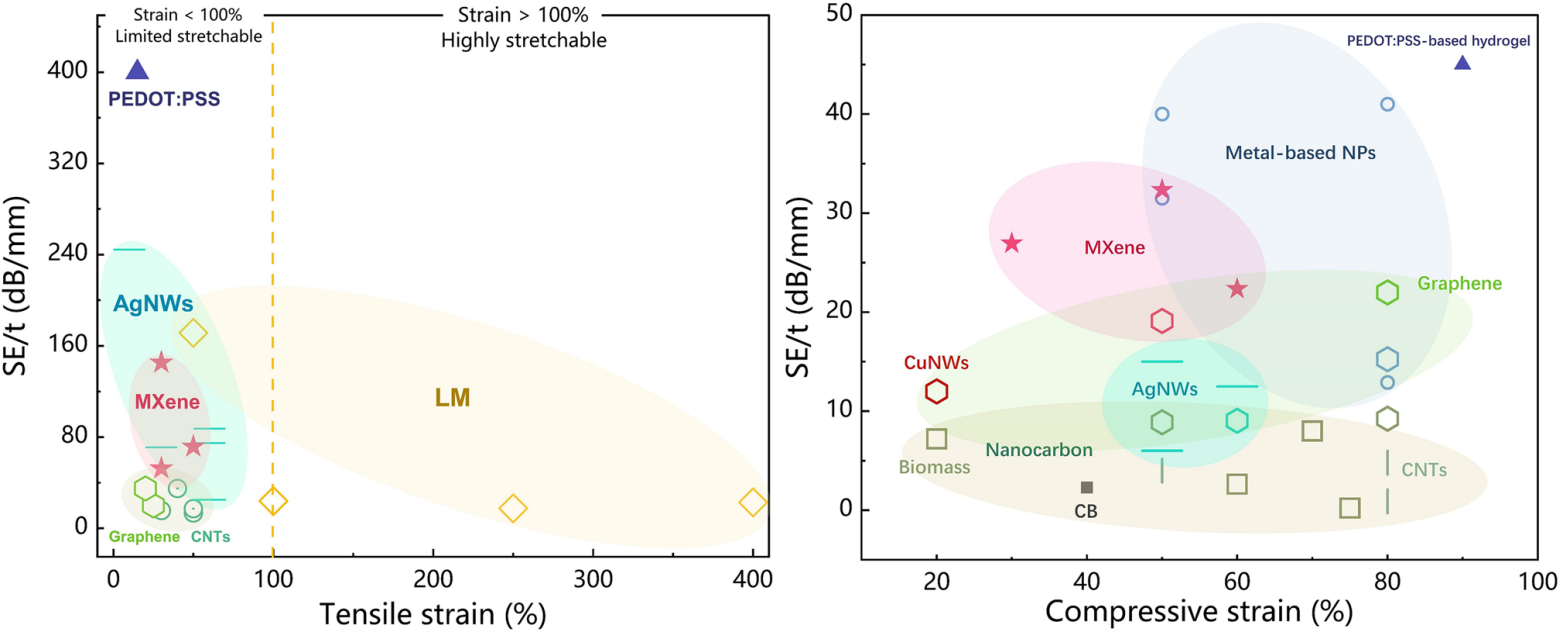
Comparison of EMI SE after numerous strain cycles and tensile/compressive strain of the elastic EMI shielding materials. Each symbol indicates a set of composites as follows: AgNWs (cyan short horizontal lines), MXenes (red solid stars), Metal-based NPs (blue open circles), LM (orange open diamonds), CNTs (green vertical lines & open circles), CB (black solid square), PEDOT:PSS (navy blue solid triangles) and graphene (green open triangles). Among them, the graphene-other functional nanofillers hybrid composites are represented by specific color hexagon, depended on hybrid nanofillers. Detailed data of each point are presented in Tables 1 and 2

For the successful development of durable and efficient EMI shielding elastomers, it is necessary to produce functional materials with high electrical conductivity and mechanical resilience. In this review, promising candidates for functional materials are evaluated in terms of fabrication techniques, pre/post-treatment, and physical properties, such as thickness, density, electrical conductivity, EM wave loss capacity, stretchability/compressibility, and inner structure. Table 1 lists the benefits and drawbacks of several functional materials used in elastic EMI shielding materials. And Fig. 14 shows the comparison of EMI SE after numerous strain cycles and tensile/compressive strain of the elastic EMI shielding materials. Below is a synopsis of where functional materials are headed and some of the challenges they face.

(i) Synergistic effect

**Table 1:** Summary of stretchable EMI shielding materials

Filler/Matrix	EMI SE			Max. stretchability (%)	Changes in EMI SE under applied strains	References
	Thickness (mm)	Conductivity	(− dB)			
AgNPs/SEBS	2.8	~ 1000 S m^−1^	55	100	~ 28 dB at 100% strain	[64]
AgNPs/SBS	–	~ 8000 S m^−1^	~ 45	60	~ 30 dB after 300-cycle at 60% strain	[63]
CB/ Chlorinated polyethylene	5	0.379 S m^−1^	~ 40^☆^	~ 100	–	[292]
AgNWs/WPU-PDMS	1	~ 180 S m^−1^	28	60	~ 25 dB after 1000-cycle at 60% strain	[29]
AgNWs/silicon elastomer	0.5	4.58 ± 0.57 Ω sq^−1^	~ 32	50	~ 22 dB at 50% strain	[144]
AgNWs/textiles-PU	0.6	1227 S m^−1^	63.9	60	~ 52.4 dB after 5000-cycle at 60% strain	[143]
AgNWs/carbon fiber fabric-PU	0.36	15,390 S m^−1^	106	15	~ 88 dB after 100-cycle at 10% strain	[145]
AgNWs/PDMS	–	3 Ω sq^−1^	~ 43	50	~ 34 dB at 50% strain	[36]
AgNWs/PDMS	1	~ 7500 S m^−1^	74.7	~ 170	SE maintained at 60% strain	[152]
AgNWs-rGO/PDMS	–	3.3 Ω sq^−1^	35.5	70	SE slightly decrease after 1000-cycle at 40% strain	[155]
AgNWs-graphene/PDMS	–	~ 12 Ω sq^−1^	37	60	~ 30 dB at 50% strain	[226]
AgNWs-CNTs/textiles	0.6	~ 530 S m^−1^	51.5	~ 200	42.6 dB after 5000-cycle at 30% strain	[293]
AgNWs-MXene/PDA polyester fabric	0.6	150 S m^−1^	44	~ 445	~ 30 dB after 1000-cycle	[294]
CNTs/PU foam-Ecoflex	2.9	~ 100 S m^−1^	~ 35	~ 100	20.2 dB at 30% strain	[123]
CNTs/TPU	2	~ 10 S m^−1^	~ 34.5	~ 240	~ 30 dB at 50% strain	[122]
CNTs/Natural rubber	2.6	~ 100 S m^−1^	~ 45	~ 200	35 dB after 5000-cycle at 50% strain	[295]
CNTs sponge/PDMS	1.8	~ 180 S m^−1^	54.8	–	SE maintained after 1000-cycle	[121]
CNTs sponge/PDMS	1	53 S m^−1^	~ 35	40	SE slightly decrease after 500-cycle	[296]
CNTs/PDMS microspheres	2.5	64.6 S m^−1^	47	~ 85	SE retention of 80% after 1000-cycle at 30% strain	[297]
CNTs-MXene/PDA-TPU fabric	0.6	~ 50 S m^−1^	43	~ 200	SE maintained after 1000-cycle at 50% strain	[240]
CNTs-MXene-GO/Latex	0.1 × 10^–2^ *	15 Ω	~ 30	~ 800 (area strain)	SE maintained after 500-cycle 800% areal strain	[184]
rGO-Fe_3_O_4_/Natural rubber	0.6	~ 1600 Ω	~ 12	75	SE maintained after 500-cycle 25% strain	[223]
rGO/PDMS lattice	4.8	~ 25 S m^−1^	~ 40	~ 130	~ 25 dB at 100% strain	[225]
rGO/woven fabric-PDMS	1.2	~ 40 S m^−1^	45	–	42 dB after 100-cycle at 20% strain	[224]
N-doping rGO/Wrinkled PDMS	0.66 × 10^–2^ *	8796 S cm^−1^	58.5	–	56.3 dB after 100-cycle at 32.6% strain	[199]
MXene/Wrinkled PDMS	0.3 × 10^–3^ *	~ 100 Ω	~ 30	100	SE maintained at 25% strain	[243]
MXene/Natural rubber	0.172	1400 S m^−1^	~ 25	200	SE maintained at 30% strain	[298]
MXene/Wrinkled TPU fabric	–	–	~ 30	70	~ 20 dB after 50-cycle at 70% strain	[241]
MXene-Fe_3_O_4_/Modified natural rubber	~ 0.571	~ 1 S m^−1^	~ 36	317	~ 30 dB after bended 140˚ and stretched 30% 1000 cycles	[299]
LM/Ecoflex	2	10^6^ S m^−1^	34.5	400	86.2 dB at 400% strain	[23]
LM/Ecoflex	3.6	10^6^ S m^−1^	57	400	85 dB at 400% strain	[291]
LM-NiNPs/Ecoflex	0.05	2.4 × 10^6^ S m^−1^	> 75	300	> 45 dB at 300% strain	[258]
LM/textiles-PDMS	0.35	1.4 × 10^5^ S m^−1^	~ 75	50	~ 60 dB after 5000-cycle at 50% strain	[260]
LM/PDMS lattice	3	1.98 × 10^6^ S m^−1^	72	180	SE maintained after 1000-cycle at 100% strain	[17]
PEDOT:PSS-Fe_3_O_4_/PVA hydrogel	1	0.31	> 45	~ 904.5	> 28 dB at 800% strain	[95]
PEDOT:PSS/WPU	0.15	7.7 × 10^3^ S m^−1^	~ 60	~ 30	SE maintained after 100-cycle at 15% strain	[268]

*The thickness of functional layer; ☆: This Reference is not tested for EMI SE in the X-band regime, while all other References are

**Table 2:** Summary of compressive EMI shielding materials

Filler/Matrix	Method	EMI SE			Elastic modulus (kPa)	Fatigue resistance			References
		h* (mm)	Conductivity (S m^−1^)	(−dB)		Cycle	Strain (%)	EMI SE	
AgNPs/MF sponge	MOD*	3.1	158.4	~ 40	~ 5	10	80	–	[66]
AgNPs/PDA-PU sponge	Electroless plating	2	1.24	82	~ 100	400	80	~ 75	[58]
AgNPs-CNTs/SBS foam	Freeze-casting	2	582	63	20.6	1000	50	~ 84	[73]
AgNP-graphene aerogel	Freeze-casting	0.8	3.2	~ 32	~ 20	10	50	–	[74]
AuNPs-graphene-Fe_3_O_4_/Carbonized MF-PDMS composites	Dip-coating	2	100	30.5	~ 50	1	80	–	[61]
Hollow CB/silicon rubber	Melt blending	–	–	~ 23^☆^	~ 3 × 10^3^	4	48	–	[81]
AgNWs/Modified MF	Roll-to-roll	10	10^4^	SE_R_ = 60	–	1000	50	58	[147]
AgNWs-MXene/MF sponge	Dip-coating	2	103	25	26	500	60	23	[146]
AgNWs-MXene/MF sponge	Dip-coating	2	75.3	~ 30	–	–	50	12.4	[287]
AgNWs-rGO aerogel	Freeze-casting	5	~ 0.2	~ 45.2	~ 62.5	10	60	–	[191]
CuNWs-graphene aerogel	Freeze-casting	5	~ 10^3^	~ 60	~ 3	100	20	–	[176]
CNTs/PU-TPI foam*	Dip-coating	10	–	~ 40	–	–	80	~ 30	[127]
						500	50	35	
CNTs/PI foam	Dip-coating	12	–	~ 57.6	~ 17	1000	80	~ 40	[125]
CNTs/Modified chitosan-PU foam	Dip-coating	40	498	~ 36	–	–	75	~ 18	[285]
						2000	80	~ 30	
rGO-Lignin derived carbon aerogel	Freeze-casting	9	~ 30	~ 80	~ 5	100	50	76	[276]
Graphene/MF-TPU foam	Dip-coating	2	45.2	~ 35	~ 400	100	–	~ 34	[32]
GO/CNF-PMMA aerogel	Freeze-casting	4	–	~ 37	~ 0.8	5000	80	–	[208]
GO aerogel	3D printing	3	705.6	~ 66	~ 55	100	80	–	[288]
rGO-CNTs/PI foam	Freeze-casting	–	~ 22.5	28.2	~ 12.5	10	50	–	[124]
MXene/Sodium alginate-PDMS foam	Freeze-casting	2	~ 800	53.9	–	500	30	48.2	[239]
MXene/Wood aerogel	Freeze-casting	10	37	72	~ 50	400	20	–	[289]
Modified MXene/ Natural wood derived carbon foam	Dip-coating	10	~ 10^−7^	~ 26.3	~ 50	–	60	~ 16.1	[248]
MXene/MF-PDMS foam	Dip-coating	2	183	44.7	~ 25	200	60	37.6	[300]
MXene/MF sponge	Dip-coating	–	~ 20	~ 5^☆^	~ 12	–	80	~ 42	[301]
MXene-CB/PANI decorated modified PP-PDMS foam	Dip-coating	12	–	~ 27.7	~ 666	–	40	~ 16.2	[302]
						500	40	~ 25.5	
MXene-rGO scaffolds	3D printing & Freeze-casting	3.4	1013	> 60	~ 2400	100	50	–	[303]
MXene-AgNWs/PU foam	Freeze-casting	1.3	~ 1100	47.4	–	1000	–	41.5	[304]
MXene-NiFe_2_O_4_/WPU aerogel	Freeze-casting	2	226.4	64.7	~ 200	100	50	–	[89]
Wheat flour-CNTs foam	Freeze-casting	5	0.1	~ 40	~ 20	–	70	~ 15	[126]
						1	70	~ 39	
Wood-derived carbon/CB aerogel	Dip-coating	6.9	0.16	~ 1.5	~ 30	–	75	~ 25.5	[21]
LM/PDMS foam	Sugar template	10	800	~ 45	~ 100	–	50	~ 80	[262]
						10^4^	50	~ 50	
PEDOT:PSS aerogel	Freeze-casting	1.4	~ 10^–4^	15	–	–	> 90	24	[271]
PEDOT:PSS-Fe_3_O_4_/PVA hydrogel	Self-assembly	1	0.31	> 45	~ 35	10	90	–	[95]

*h thickness; *MF* melamine foam; *TPI* trans-1,4-polyisoprene; ☆: This Reference is not tested for EMI SE in the X-band regime, while all other References are; –: Only compress, not release

Most functional materials, as shown in Table 3, have advantages and disadvantages. As a result, both in terms of EMI SE and mechanics, single material architectures are challenging barriers for EMI shielding materials to overcome. However, the synergy of one of the fillers with another filler with widely varying properties to construct an elastic EMI shielding material can sometimes effectively solve the problems that exist with a single material, thus improving the performance of the material as a whole. For example, the AgNPs-graphene and/or CNTs system not only effectively solves the problem of high use and consumption of AgNPs, but also enhances the electrical conductivity exhibited by the nanocarbon, thus effectively improving the EMI SE of the material while reducing costs. In addition, the AgNWs or CuNWs-MXene system also effectively enhances the chemical and environmental stability of metal nanowires through graphene and/or MXene encapsulation by combining the tensile advantages of 1D materials with the large specific surface of 2D materials. And this initially resolves the issue of insufficient tensile properties of 2D materials by forming “island-bridge” structure. Notably, the most crucial synergistic strategy is to mix magnetic fillers with highly conductive fillers in order to jointly impose magnetic losses and conduction losses, which can further increase the EMI shielding performance in a way that neither filler can do on its own. Thus, the synergistic impact of “1 + 1 > 2” may be achieved by the effective combination of diverse functional materials, but this topic still has to be studied systematically in order to reach its full potential.

(ii) Elasticity

In the previous analysis, for stretchable EMI films, for the formation of percolation networks to conduct electricity nanofillers can effectively enhance the material stretching performance, but their partial local conductive networks break as the stretching range increases, which can make the EMI shielding performance degraded, or even the overall break [277]. At the same time, during extremely large-scale stretching, the nanofillers are utterly unable to sustain the EMI shielding capability. At present, only LM can achieve more than 400% effective stretching with high EMI shielding due to its own characteristics, which break the bondage of the percolation network. Thus, one of the hottest areas of study in the future will be the hunt for the creation of novel functional materials capable of large-scale stretching. For compressive materials, the biggest challenge is how to achieve structural integrity and stable EMI shielding performance after large-scale, multi-cycle compression of compressible materials.

**Table 3:** Comparison of different functional materials-based EMI shielding materials

	Functional materials	Advantages		Disadvantages
0D	AgNPs	Easy preparation; ultrahigh conductivity; broad antibacterial spectrum		Poor chemical stability; large mass consumption
	Carbon Black	Abundant carbon resources; large-scale production		Poor conductivity; harmfulness; large mass consumption
	Ferrites	Excellent magnetic loss performance; good dielectric properties		Poor conductivity; Poor stretchability
	Transition metal	Excellent magnetic loss performance; high conductivity		Poor stretchability;
1D	AgNWs	Ultrahigh conductivity; broad antibacterial spectrum; high transparency; good deformability; high EMI shielding ability		Post-treatment required; Poor chemical & thermal stability
CuNWs		High conductivity; high transparency; good deformability; cheaper cost		Post-treatment required; Poor chemical stability
	CNTs	Abundant carbon resources; high transparency; good deformability		low conductivity
2D	Graphene	Abundant carbon resources; high transparency; low surface roughness; high chemical stability		low conductivity; post-treatment required; Poor stretchability
	MXene	ultrahigh conductivity; low surface roughness; ultrahigh EMI shielding ability; easily modified		Poor environmental stability; Poor stretchability; Cumbersome preparation; Waste generated
Others	Liquid metal	Ultrahigh conductivity; superb deformability; ultrahigh EMI shielding ability		Leakage
	Conductive polymer	High conductivity; excellent biocompatibility; good durability; good environmental stability		Poor stretchability; low chemical stability
	Biomass carbon	Renewable resources; good environmental friendliness; easy preparation		ultralow conductivity; poor EMI shielding ability

As far as we can see, it is difficult for the compression/tension behavior not to have an impact on the EMI shielding performance. Therefore, the intelligent use of changes in EMI shielding performance, resulting in a reconfigurable “on–off” smart EMI shielding film. And one of the most significant issues that radar and antenna transmitting systems face today is how to provide essential signals while adequately protecting against EMI interference.

(iii) Stability

Currently, the majority of available EMI shielding films have already attained extremely effective shielding against EMI interference. However, there is not enough thorough and organized study on EMI shielding stability, which is required for the EMI shielding film to be marketed. Besides, it is an efficient strategy to capsulate functional components for effectively isolating them from oxygen and water due to the polymer matrix’s typically strong durability. Based on this, the encapsulation of metal nanowires using nanomaterials such as graphene is also one of the significant methods [155]. While improving the thermal stability as well as chemical stability of metal nanowires, it does not cause a significant increase in the overall material weight [278].

(iv) Multifunction

For different application scenarios, individuals prefer EMI shielding films to have other functions as well. For wearable devices, accurate sensing is essential to monitor human activity. And the ability to dissipate accumulated heat in time maximizes the comfort of the device. The rearrangement of conductive filler alignment due to thin film deformation has a significant impact on the heat dissipation of elastic EMI shielding films, which can be further utilized for thermal management [279]. At the same time, for scenarios such as the Internet of Things, the right transparency can be used without compromising the perception of the original item. The development of multifunctional, elastic EMI shielding materials will therefore enable a further expansion of their application.

In general, such elastic materials with unique machanical properties will be the potential candidates in the field of EMI shielding in the future. We expect that this review will offer a thorough understanding of the obstacles and potential future development of unique elastomer shields as well as present more fresh possibilities for the development of next-generation EMI shielding materials.

## Abbreviations

EMI: Electromagnetic interference
EM: Electromagnetic
SoC: System-on-chips
SIP: System-in-package
RFIC: Radio frequency integrated circuits
SE: Shielding efficiency
CNTs: Carbon nanotubes
AgNPs: Silver nanoparticles
AuNPs: Gold nanoparticles
CB: Carbon black
AgNWs: Silver nanowires
CuNWs: Copper nanowires
SE_T_: The total SE of EMI
SE_R_: Reflection loss
SE_A_: Absorption loss
SE_M_: Multiple reflection loss
R_sq_: Square resistance
FL: Few-layer structure
ML: Multilayer structure
MF: Melamine foam
TPI: Trans-1,4-polyisoprene
PU: Polyurethane
PDMS: Poly(dimethyl siloxane)
SBS: Poly(styrene-butadiene-styrene)
SEBS: Poly(styrene-co-ethyl-enebutylene-co-styrene)
CNF: Cellulose nanofibers
NCG: Nickel-coated graphite
HCB: Hollow carbon black
WPU: Waterborne polyurethane
PEDOT:PSS: Poly(3,4-ethylenedioxythiophene)-poly(styrene sulfonic acid)
PVA: Polyvinyl alcohol
PET: Polyester
SWCNTs: Single-walled carbon nanotubes
MWCNTs: Multiwalled carbon nanotubes
PIFs: Polyimide foams
WF: Wheat flour
HPMC: Hydroxypropyl methylcellulose
CVD: Chemical vapor deposition
rGO: Reduced graphene oxide
GO: Graphene oxide
CNH: Carbon nanohorn
HMTA: Hexamethylenetetramine
PMMA: Polymethyl methacrylate
ANF: Aramid nanofiber
GNSs: Graphene nanosheets
NR: Natural rubber
GNR: Graphene nanoribbon
LM: Liquid metals
SA: Sodium alginate
PPy: Polypyrrole
